# Supplementary material for: Intraocular pressure elevation after subtenon triamcinolone acetonide injection; Multicentre retrospective cohort study in Japan
Source: PLoS One. 2019 Dec 5;14(12):e0226118. doi: 10.1371/journal.pone.0226118 (PMC6894825; doi:10.1371/journal.pone.0226118)
Supplement: S1 Dataset — (PDF) [file pone.0226118.s001.pdf]

Eye No	Pt No	age	M=0,F=1	R=0,L=1	BLIOP	R=0,D=1,u=2,o=3	上昇(+)=0,(-)=1	mg	STTAtime	day	highestIOP	眼圧差	眼圧6以上の変化あり Yes=0, No =1	Medication=0,Surgical procedure=1,no treatment=2	Bilateral STTA=0, unilateral STTA=1
1	1	66	0	0	12	1	1	20	1		15	3	1		0
2	1	66	0	1	13	1	1	20	1		17	4	1		0
3	2	71	1	1	12	1	1	20	1		16	4	1		1
4	3	68	1	1	16	1	1	20	1		19	3	1		1
5	4	42	0	1	13	1	1	20	1		16	3	1		0
6	4	42	0	0	14	1	1	20	1		16	2	1		0
7	5	78	1	1	14	0	1	20	1		18	4	1		1
8	6	52	0	1	12	1	1	20	1		14	2	1		1
9	7	65	0	1	14	1	1	20	1		14	0	1		0
10	7	65	0	0	10	1	1	20	1		12	2	1		0
11	8	39	0	1	20	1	1	20	1		21	1	1		1
12	9	67	0	1	14	1	1	20	1		17	3	1		1
13	10	66	1	1	11	1	1	20	1		18	7	0		0
14	10	66	1	0	13	1	1	20	1		18	5	1		0
15	11	83	1	1	14	1	1	20	1		17	3	1		1
16	12	64	0	0	15	1	0	20	1	81	27	12	0	2	1
17	13	62	0	1	18	1	0	20	1	1	22	4	1	2	1
18	14	83	1	0	5	2	1	20	1		10	5	1		1
19	15	67	0	0	18	1	0	20	1	32	23	5	1	2	0
20	15	67	0	1	18	1	0	20	1	46	29	11	0	2	0
21	16	59	0	1	14	1	1	20	1		17	3	1		0
22	16	59	0	0	15	1	1	20	1		16	1	1		0
23	17	73	1	1	12	1	1	20	1		14	2	1		1
24	18	68	0	0	11	1	1	20	1		15	4	1		0
25	18	68	0	1	13	1	1	20	1		17	4	1		0
26	19	66	0	1	20	1	1	20	1		20	0	1		1
27	20	66	1	1	16	1	1	20	1		17	1	1		0
28	20	66	1	0	11	1	1	20	1		19	8	0		0
29	21	67	0	1	12	1	1	20	1		18	6	0		1
30	22	48	0	1	16	1	1	20	1		20	4	1		0
31	22	48	0	0	16	1	1	20	1		20	4	1		0
32	23	61	0	1	10	3	1	20	1		12	2	1		1
33	24	40	0	1	17	1	1	20	1		20	3	1		1
34	25	70	0	1	18	1	1	20	1		18	0	1		1
35	26	71	1	0	13	1	1	20	1		12	-1	1		0
36	26	71	1	1	13	1	1	20	1		17	4	1		0
37	27	72	0	0	14	1	1	20	1		19	5	1		1
38	28	68	0	0	16	1	1	20	1		19	3	1		1
39	29	59	1	0	16	3	1	20	1		18	2	1		1
40	30	85	1	1	12	1	1	20	1		12	0	1		1
41	31	59	0	1	14	0	1	20	1		17	3	1		1
42	32	70	1	0	18	1	1	20	1		18	0	1		1
43	33	38	1	0	17	0	0	20	1	28	36	19	0	0	1
44	34	59	0	0	14	1	1	20	1		17	3	1		1
45	35	61	0	1	18	0	0	20	1	59	24	6	0	2	1
46	36	48	0	1	17	3	0	20	1	14	22	5	1	0	1
47	37	59	0	0	16	1	1	20	1		20	4	1		1
48	38	65	0	1	18	1	1	20	1		19	1	1		1
49	39	70	0	0	16	1	1	20	1		17	1	1		0
50	39	70	0	1	16	1	1	20	1		17	1	1		0
51	40	70	0	1	20	1	1	20	1		20	0	1		1
52	41	32	0	1	13	1	1	20	1		18	5	1		1
53	42	73	1	0	13	1	1	20	1		13	0	1		0
54	42	73	1	1	14	1	1	20	1		14	0	1		0
55	43	70	0	0	12	1	1	20	1		16	4	1		1
56	44	55	0	0	13	1	1	20	1		17	4	1		1
57	45	71	1	0	12	1	1	20	1		14	2	1		1
58	46	43	0	1	14	1	1	20	1		17	3	1		1
59	47	71	0	0	14	1	1	20	1		18	4	1		1
60	48	57	0	0	12	0	1	20	1		17	5	1		1
61	49	66	1	1	17	0	1	20	1		18	1	1		1
62	50	65	0	0	12	0	1	20	1		19	7	0		1
63	51	74	0	1	13	0	1	20	1		17	4	1		1
64	52	72	0	0	16	1	1	20	1		20	4	1		1
65	53	65	0	0	14	1	1	20	1		17	3	1		0
66	53	65	0	1	14	1	1	20	1		16	2	1		0
67	54	70	0	1	12	0	1	20	1		12	0	1		1
68	55	62	0	1	12	1	1	20	1		16	4	1		1
69	56	83	1	0	14	1	1	20	1		14	0	1		0
70	56	83	1	1	14	1	1	20	1		14	0	1		0
71	57	65	0	0	16	1	1	20	1		18	2	1		1
72	58	68	0	0	12	0	1	20	1		14	2	1		0
73	58	68	0	1	18	1	0	20	1	44	31	13	0	0	0
74	59	72	1	1	13	0	1	20	1		14	1	1		1
75	60	66	0	0	13	1	1	20	1		21	8	0		0
76	60	66	0	1	17	1	1	20	1		15	-2	1		0
77	61	66	0	0	14	1	1	20	1		18	4	1		1
78	62	74	0	0	11	1	1	20	1		15	4	1		0
79	62	74	0	1	14	1	1	20	1		14	0	1		0
80	63	78	0	0	10	0	1	20	1		10	0	1		1
81	64	78	0	0	8	1	1	20	1		13	5	1		1
82	65	65	0	0	16	0	1	20	1		18	2	1		1
83	66	76	1	0	15	0	1	20	1		18	3	1		1
84	67	67	0	1	16	0	1	20	1		16	0	1		1
85	68	56	1	1	16	0	1	20	1		16	0	1		1
86	69	63	0	1	14	1	1	20	1		16	2	1		1
87	70	63	1	0	18	1	0	20	1	36	26	8	0	2	1
88	71	69	0	0	16	0	1	20	1		17	1	1		1
89	72	60	0	1	14	1	1	20	1		14	0	1		1
90	73	68	0	0	16	0	1	20	1		18	2	1		1
91	74	70	0	1	18	1	1	20	1		21	3	1		1
92	75	84	1	1	17	0	1	20	1		21	4	1		1
93	76	70	0	0	16	1	1	20	1		18	2	1		0
94	76	70	0	1	18	1	1	20	1		13	-5	1		0
95	77	67	1	0	11	1	1	20	1		11	0	1		1
96	78	63	0	1	14	1	1	20	1		16	2	1		0
97	78	63	0	0	16	1	1	20	1		17	1	1		0
98	79	69	1	0	11	0	1	20	1		14	3	1		1
99	80	75	0	0	11	1	1	20	1		11	0	1		1
100	81	73	1	0	16	1	1	20	1		17	1	1		1
101	82	76	0	0	16	0	1	20	1		16	0	1		1
102	83	76	0	0	13	0	1	20	1		14	1	1		0
103	83	76	0	1	12	0	1	20	1		14	2	1		0
104	84	70	0	1	18	0	1	20	1		20	2	1		1
105	85	63	0	0	12	1	0	20	1	14	28	16	0	0	0
106	85	63	0	1	18	1	0	20	1	21	30	12	0	0	0
107	86	45	1	0	14	1	1	20	1		20	6	0		0
108	86	45	1	1	14	1	1	20	1		18	4	1		0
109	87	87	1	0	11	0	1	20	1		14	3	1		1
110	88	63	0	0	16	1	1	20	1		18	2	1		1
111	89	74	1	0	14	1	1	20	1		19	5	1		1
112	90	55	0	0	16	0	1	20	1		18	2	1		0
113	90	55	0	1	16	0	1	20	1		18	2	1		0
114	91	35	0	1	18	1	1	20	1		21	3	1		1
115	92	58	1	0	16	0	1	20	1		16	0	1		1
116	93	77	1	0	13	3	1	20	1		16	3	1		1
117	94	61	1	0	17	0	1	20	1		17	0	1		0
118	94	61	1	1	17	0	1	20	1		17	0	1		0
119	95	78	0	1	16	0	1	20	1		17	1	1		1
120	96	81	0	1	10	1	1	20	1		14	4	1		1
121	97	66	0	1	17	1	1	20	1		17	0	1		1
122	98	70	0	1	12	1	1	20	1		12	0	1		1
123	99	74	1	1	13	1	1	20	1		16	3	1		1
124	100	78	0	0	8	0	1	20	1		13	5	1		1
125	101	80	1	1	18	1	1	20	1		18	0	1		1
126	102	63	0	1	18	1	0	20	1	1	24	6	0	2	0
127	102	63	0	0	19	0	1	20	1		20	1	1		0
128	103	53	1	1	15	1	1	20	1		20	5	1		1
129	104	57	0	1	16	3	1	20	1		16	0	1		1
130	105	36	1	0	16	1	1	20	1		20	4	1		1
131	106	74	1	0	18	0	1	20	1		14	-4	1		1
132	107	53	0	1	14	1	1	20	1		15	1	1		1
133	108	69	0	0	16	0	1	20	1		17	1	1		1
134	109	65	0	1	15	1	0	20	1	26	32	17	0	0	1
135	110	64	0	0	17	1	1	20	1		17	0	1		0
136	110	64	0	1	12	1	1	20	1		18	6	0		0
137	111	82	0	0	14	0	1	20	1		13	-1	1		1
138	112	44	1	1	18	0	1	20	1		20	2	1		1
139	113	57	1	0	18	0	1	20	1		20	2	1		1
140	114	69	0	1	13	0	1	20	1		12	-1	1		1
141	115	82	0	1	14	1	1	20	1		18	4	1		1
142	116	57	0	0	16	1	1	20	1		13	-3	1		1
143	117	60	0	0	16	1	1	20	1		14	-2	1		1
144	118	61	0	1	15	1	1	20	1		15	0	1		1
145	119	68	0	1	13	0	1	20	1		13	0	1		1
146	120	64	0	0	18	0	0	20	1	128	26	8	0	0	1
147	121	74	0	0	10	0	1	20	1		10	0	1		0
148	121	74	0	1	10	1	1	20	1		10	0	1		0
149	122	56	0	1	9	0	1	20	1		9	0	1		0
150	122	56	0	0	11	0	1	20	1		14	3	1		0
151	123	46	0	1	18	0	0	20	1	104	23	5	1	0	1
152	124	77	0	0	8	0	1	20	1		13	5	1		1
153	125	69	0	1	12	0	1	20	1		17	5	1		1
154	126	49	0	0	14	1	1	20	1		19	5	1		1
155	127	71	0	0	10	0	1	20	1		11	1	1		1
156	128	80	1	1	17	0	1	20	1		16	-1	1		0
157	128	80	1	0	18	0	1	20	1		20	2	1		0
158	129	48	1	1	12	1	1	20	1		16	4	1		1
159	130	71	1	0	20	1	1	20	1		20	0	1		0
160	130	71	1	1	19	0	1	20	1		20	1	1		0
161	131	56	0	0	18	0	1	20	1		20	2	1		0
162	131	56	0	1	19	0	1	20	1		18	-1	1		0
163	132	67	1	0	10	0	1	20	1		14	4	1		0
164	132	67	1	1	12	3	1	20	1		18	6	0		0
165	133	69	1	0	11	0	1	20	1		14	3	1		1
166	134	41	0	0	16	1	1	20	1		20	4	1		1
167	135	52	0	1	16	0	1	20	1		16	0	1		1
168	136	61	1	0	12	1	1	20	1		18	6	0		1
169	137	54	0	1	17	0	1	20	1		18	1	1		1
170	138	66	0	0	10	0	1	20	1		17	7	0		1
171	139	62	1	1	17	0	0	20	1	42	24	7	0	0	0
172	139	62	1	0	20	0	1	20	1		20	0	1		0
173	140	58	0	1	13	1	1	20	1		18	5	1		1
174	141	67	0	1	14	1	1	20	1		16	2	1		0
175	141	67	0	0	14	1	1	20	1		16	2	1		0
176	142	72	1	0	9	1	1	20	1		13	4	1		1
177	143	70	1	0	17	1	1	20	1		18	1	1		1
178	144	65	0	0	14	1	1	20	1		14	0	1		1
179	145	55	0	1	14	0	1	20	1		16	2	1		1
180	146	58	1	0	17	1	0	20	1	49	23	6	0	2	1
181	147	67	1	0	12	0	1	20	1		12	0	1		1
182	148	59	0	1	17	1	1	20	1		21	4	1		1
183	149	56	0	1	20	0	1	20	1		16	-4	1		1
184	150	85	1	0	16	0	1	20	1		16	0	1		1
185	151	70	0	0	13	1	1	20	1		17	4	1		1
186	152	40	0	1	15	0	1	20	1		13	-2	1		1
187	153	63	0	0	14	0	1	20	1		18	4	1		0
188	153	63	0	1	16	1	1	20	1		20	4	1		0
189	154	76	1	1	14	0	1	20	1		17	3	1		0
190	154	76	1	0	15	0	1	20	1		18	3	1		0
191	155	52	0	1	15	0	1	20	1		18	3	1		1
192	156	50	0	0	13	1	0	20	1	56	26	13	0	0	0
193	156	50	0	1	13	1	1	20	1		15	2	1		0
194	157	66	0	0	13	1	1	20	1		14	1	1		1
195	158	68	0	0	13	1	1	20	1		16	3	1		1
196	159	68	1	1	12	0	1	20	1		14	2	1		1
197	160	24	1	0	17	1	1	20	1		17	0	1		0
198	160	24	1	1	17	1	1	20	1		17	0	1		0
199	161	39	0	1	17	1	0	20	1	144	28	11	0	0	0
200	161	39	0	0	17	1	0	20	1	137	27	10	0	0	0
201	162	64	0	0	10	1	1	20	1		14	4	1		1
202	163	69	0	0	13	1	1	20	1		18	5	1		0
203	163	69	0	1	12	1	1	20	1		18	6	0		0
204	164	75	1	1	15	1	1	20	1		16	1	1		1
205	165	66	0	1	14	1	1	20	1		14	0	1		0
206	165	66	0	0	14	1	1	20	1		18	4	1		0
207	166	65	1	1	17	3	1	20	1		19	2	1		1
208	167	67	1	1	12	0	1	20	1		15	3	1		1
209	168	71	0	0	16	1	1	20	1		20	4	1		0
210	168	71	0	1	16	1	1	20	1		13	-3	1		0
211	169	70	1	0	13	0	1	20	1		13	0	1		0
212	169	70	1	1	13	0	1	20	1		13	0	1		0
213	170	54	0	0	14	0	0	20	1	66	28	14	0	0	1
214	171	51	0	0	15	1	1	20	1		19	4	1		0
215	171	51	0	1	18	1	0	20	1	28	26	8	0	0	0
216	172	74	0	1	16	1	1	20	1		18	2	1		1
217	173	70	1	0	14	3	1	20	1		15	1	1		1
218	174	90	0	0	17	0	1	20	1		20	3	1		1
219	175	72	0	1	16	1	0	20	1	22	30	14	0	0	1
220	176	90	1	0	10	3	1	20	1		14	4	1		1
221	177	80	1	0	15	0	1	20	1		19	4	1		1
222	178	85	0	0	16	1	1	20	1		18	2	1		1
223	179	81	1	0	10	0	1	20	1		13	3	1		1
224	180	73	1	0	19	1	1	20	1		21	2	1		0
225	180	73	1	1	16	1	1	20	1		18	2	1		0
226	181	68	1	0	14	3	1	20	1		19	5	1		1
227	182	73	1	1	15	0	1	20	1		18	3	1		1
228	183	33	0	1	16	1	0	20	1	61	22	6	0	2	1
229	184	65	0	1	14	3	1	20	1		17	3	1		1
230	185	35	0	0	20	1	1	20	1		19	-1	1		0
231	185	35	0	1	16	1	1	20	1		18	2	1		0
232	186	30	0	0	15	1	1	20	1		21	6	0		0
233	186	30	0	1	17	1	1	20	1		20	3	1		0
234	187	66	0	1	13	2	1	20	1		13	0	1		1
235	188	73	0	1	12	1	1	20	1		19	7	0		1
236	189	72	1	0	14	3	1	20	1		18	4	1		1
237	190	89	1	0	18	2	1	20	1		18	0	1		0
238	190	89	1	1	11	2	1	20	1		14	3	1		0
239	191	82	1	1	11	1	1	20	1		12	1	1		1
240	192	77	1	0	8	2	1	20	1		12	4	1		0
241	192	77	1	1	11	2	1	20	1		13	2	1		0
242	193	54	1	0	12	1	1	20	1		15	3	1		1
243	194	75	1	0	15	1	1	20	1		18	3	1		1
244	195	55	0	0	14	0	0	20	1	32	28	14	0	2	1
245	196	49	0	0	13	1	1	20	1		15	2	1		0
246	196	49	0	1	13	1	1	20	1		15	2	1		0
247	197	93	1	0	14	0	1	20	1		18	4	1		1
248	198	70	1	0	14	0	1	20	1		19	5	1		1
249	199	65	1	0	12	2	0	20	1	52	32	20	0	0	1
250	200	75	0	0	12	1	1	20	1		18	6	0		0
251	200	75	0	1	12	1	1	20	1		15	3	1		0
252	201	61	0	1	12	0	1	20	1		19	7	0		1
253	202	64	1	0	13	2	1	20	1		15	2	1		0
254	202	64	1	1	11	2	1	20	1		16	5	1		0
255	203	74	0	1	17	1	1	20	1		18	1	1		1
256	204	75	0	1	18	2	1	20	1		20	2	1		1
257	205	83	0	1	13	1	1	20	1		18	5	1		1
258	206	78	1	0	12	0	1	20	1		21	9	0		1
259	207	54	0	0	11	2	1	20	1		18	7	0		0
260	207	54	0	1	14	2	1	20	1		19	5	1		0
261	208	75	1	1	10	3	1	20	1		15	5	1		1
262	209	53	0	1	14	0	1	20	1		16	2	1		1
263	210	71	0	1	16	1	1	20	1		18	2	1		1
264	211	69	1	0	17	0	1	20	1		19	2	1		1
265	212	62	0	1	10	1	1	20	1		13	3	1		1
266	213	66	0	1	17	1	1	20	1		20	3	1		1
267	214	51	0	0	16	1	1	20	1		19	3	1		1
268	215	71	1	0	16	2	1	20	1		19	3	1		0
269	215	71	1	1	14	2	1	20	1		20	6	0		0
270	216	75	1	0	18	2	1	20	1		18	0	1		1
271	217	77	0	0	9	0	1	20	1		11	2	1		1
272	218	72	1	0	17	0	1	20	1		18	1	1		1
273	219	39	1	0	9	2	1	20	1		19	10	0		0
274	219	39	1	1	15	2	1	20	1		16	1	1		0
275	220	76	0	0	10	0	1	20	1		12	2	1		1
276	221	75	0	0	9	3	1	20	1		11	2	1		1
277	222	62	0	1	15	1	1	20	1		20	5	1		1
278	223	67	1	0	15	2	1	20	1		18	3	1		0
279	223	67	1	1	16	2	1	20	1		19	3	1		0
280	224	60	1	0	15	1	1	20	1		16	1	1		0
281	224	60	1	1	15	1	1	20	1		17	2	1		0
282	225	75	1	1	14	0	1	20	1		19	5	1		1
283	226	36	1	1	10	3	1	20	1		19	9	0		1
284	227	74	0	0	14	1	1	20	1		20	6	0		0
285	227	74	0	1	16	1	1	20	1		21	5	1		0
286	228	85	1	0	18	1	1	20	1		20	2	1		1
287	229	84	1	0	18	1	1	20	1		20	2	1		1
288	230	80	0	1	12	1	1	20	1		21	9	0		1
289	231	65	0	0	14	1	1	20	1		17	3	1		1
290	232	58	1	0	18	2	1	20	1		20	2	1		1
291	233	70	1	1	12	1	1	20	1		17	5	1		1
292	234	70	0	1	14	1	1	20	1		15	1	1		1
293	235	79	1	0	13	2	1	20	1		16	3	1		0
294	235	79	1	1	15	2	1	20	1		17	2	1		0
295	236	34	1	1	12	2	1	20	1		19	7	0		1
296	237	76	0	0	12	0	1	20	1		15	3	1		1
297	238	59	0	0	14	1	1	20	1		16	2	1		1
298	239	56	0	0	17	1	1	20	1		17	0	1		1
299	240	51	0	1	17	1	1	20	1		20	3	1		1
300	241	44	0	1	10	2	1	20	1		14	4	1		1
301	242	88	0	0	11	0	1	20	1		11	0	1		1
302	243	55	0	0	17	1	0	20	1	103	28	11	0	0	0
303	243	55	0	1	17	1	0	20	1	110	24	7	0	0	0
304	244	56	1	0	16	1	1	20	1		18	2	1		0
305	244	56	1	1	17	1	1	20	1		19	2	1		0
306	245	52	0	0	18	1	1	20	1		21	3	1		0
307	245	52	0	1	19	1	1	20	1		19	0	1		0
308	246	78	0	0	12	1	1	20	1		21	9	0		1
309	247	68	1	1	12	1	1	20	1		15	3	1		1
310	248	80	0	0	8	0	1	20	1		11	3	1		1
311	249	37	1	0	15	1	1	20	1		19	4	1		0
312	249	37	1	1	17	1	1	20	1		20	3	1		0
313	250	66	1	1	17	2	1	20	1		19	2	1		1
314	251	71	0	0	14	1	1	20	1		15	1	1		0
315	251	71	0	1	16	1	1	20	1		14	-2	1		0
316	252	72	1	0	14	1	1	20	1		20	6	0		1
317	253	69	0	1	12	0	1	20	1		15	3	1		1
318	254	57	0	0	13	1	1	20	1		19	6	0		1
319	255	52	1	0	13	1	1	20	1		15	2	1		1
320	256	52	1	0	17	1	1	20	1		18	1	1		1
321	257	74	0	0	14	0	1	20	1		17	3	1		1
322	258	57	0	0	10	0	1	20	1		18	8	0		1
323	259	82	1	1	14	0	1	20	1		19	5	1		1
324	260	61	0	0	14	1	1	20	1		17	3	1		0
325	260	61	0	1	12	1	1	20	1		16	4	1		0
326	261	61	1	0	16	1	1	20	1		18	2	1		0
327	261	61	1	1	15	1	1	20	1		18	3	1		0
328	262	49	0	1	13	1	1	20	1		18	5	1		1
329	263	92	1	1	11	1	1	20	1		19	8	0		1
330	264	67	1	0	18	1	1	20	1		21	3	1		0
331	264	67	1	1	17	1	1	20	1		19	2	1		0
332	265	45	1	0	19	1	1	20	1		19	0	1		0
333	265	45	1	1	18	1	1	20	1		19	1	1		0
334	266	79	1	1	9	0	1	20	1		10	1	1		1
335	267	74	1	0	14	1	1	20	1		15	1	1		1
336	268	79	1	1	13	1	1	20	1		18	5	1		1
337	269	82	0	0	19	1	1	20	1		19	0	1		1
338	270	62	1	0	19	1	0	20	1	10	24	5	1	2	1
339	271	79	1	1	10	0	1	20	1		14	4	1		1
340	272	77	0	1	8	0	1	20	1		12	4	1		1
341	273	69	1	0	12	1	1	20	1		17	5	1		1
342	274	69	1	0	12	1	1	20	1		17	5	1		1
343	275	61	0	0	14	3	1	20	1		19	5	1		1
344	276	78	0	0	11	0	1	20	1		11	0	1		1
345	277	81	0	1	15	3	1	20	1		11	-4	1		0
346	277	81	0	0	11	3	1	20	1		19	8	0		0
347	278	59	1	0	11	1	1	20	1		12	1	1		1
348	279	68	0	1	15	1	1	20	1		18	3	1		1
349	280	71	0	0	14	1	1	20	1		17	3	1		1
350	281	76	0	0	17	3	1	20	1		18	1	1		0
351	281	76	0	1	18	3	1	20	1		19	1	1		0
352	282	65	0	0	14	1	1	20	1		13	-1	1		1
353	283	62	0	1	14	1	1	20	1		16	2	1		1
354	284	69	0	1	14	1	1	20	1		14	0	1		1
355	285	59	1	0	15	2	1	20	1		18	3	1		1
356	286	58	0	1	10	1	1	20	1		12	2	1		1
357	287	62	0	0	10	1	1	20	1		18	8	0		1
358	288	55	0	1	16	1	0	20	1	98	23	7	0	2	1
359	289	49	0	1	15	0	0	20	1	66	33	18	0	0	1
360	290	64	0	0	11	1	1	20	1		20	9	0		1
361	291	77	1	0	10	0	1	20	1		12	2	1		1
362	292	73	1	1	13	0	1	20	1		15	2	1		1
363	293	65	0	1	15	1	1	20	1		17	2	1		1
364	294	83	0	1	8	0	1	20	1		14	6	0		1
365	295	66	0	1	17	3	1	20	1		20	3	1		1
366	296	60	0	1	16	1	1	20	1		20	4	1		1
367	297	59	1	0	16	3	1	20	1		19	3	1		1
368	298	49	1	0	17	2	0	20	1	20	29	12	0	2	1
369	299	49	0	1	12	2	0	20	1	97	25	13	0	2	1
370	300	64	1	1	13	0	1	20	1		14	1	1		1
371	301	80	0	1	8	3	1	20	1		16	8	0		1
372	302	73	1	0	14	0	1	20	1		18	4	1		1
373	303	53	1	0	16	3	0	20	1	44	25	9	0	2	1
374	304	66	0	0	18	1	1	20	1		21	3	1		1
375	305	36	1	0	17	1	0	20	1	83	33	16	0	0	1
376	306	57	0	0	19	1	0	20	1	121	24	5	1	2	1
377	307	66	1	0	14	2	1	20	1		16	2	1		0
378	307	66	1	1	15	2	1	20	1		16	1	1		0
379	308	49	0	0	12	3	0	20	1	54	36	24	0	0	1
380	309	70	1	0	12	2	1	20	1		15	3	1		0
381	309	70	1	1	10	2	1	20	1		16	6	0		0
382	310	63	0	0	15	1	1	20	1		13	-2	1		1
383	311	62	1	1	11	1	1	20	1		14	3	1		1
384	312	38	0	0	15	1	1	20	1		18	3	1		1
385	313	73	1	0	11	3	1	20	1		15	4	1		0
386	313	73	1	1	13	3	1	20	1		14	1	1		0
387	314	61	1	0	17	2	0	20	1	37	23	6	0	2	0
388	314	61	1	1	12	2	1	20	1		17	5	1		0
389	315	58	0	1	15	1	1	20	1		18	3	1		1
390	316	75	0	0	12	3	0	20	1	88	26	14	0	0	1
391	317	72	0	1	9	1	1	20	1		13	4	1		1
392	318	73	1	1	17	0	1	20	1		19	2	1		1
393	319	64	1	1	15	1	0	20	1	27	27	12	0	0	1
394	320	42	0	0	15	1	0	20	1	50	22	7	0	0	0
395	320	42	0	1	17	1	0	20	1	58	30	13	0	0	0
396	321	62	1	1	10	1	1	20	1		13	3	1		1
397	322	69	1	1	18	0	1	20	1		20	2	1		1
398	323	69	1	0	10	2	1	20	1		17	7	0		1
399	324	67	1	1	11	0	1	20	1		17	6	0		1
400	325	50	0	1	19	0	0	20	1	89	22	3	1	2	1
401	326	27	0	0	14	3	0	20	1	117	22	8	0	2	1
402	327	74	0	1	15	1	1	20	1		18	3	1		1
403	328	62	1	1	17	1	0	20	1	150	38	21	0	0	1
404	329	83	1	0	12	0	1	20	1		15	3	1		1
405	330	59	1	0	10	1	0	20	1	196	22	12	0	2	1
406	331	80	0	0	13	3	1	20	1		14	1	1		1

407	332	72	1	0	10	0	1	20	1		16	6	0	
408	333	49	0	0	15	1	1	20	1		20	5	1	
409	333	49	0	1	12	1	1	20	1		20	8	0	
410	334	77	1	1	18	0	0	20	1	3	23	5	1	2
411	335	89	1	0	14	0	1	20	1		16	2	1	
412	336	47	0	1	13	0	1	20	1		18	5	1	
413	337	74	0	1	12	0	1	20	1		15	3	1	
414	338	82	1	0	15	0	1	20	1		18	3	1	
415	339	89	0	0	11	0	1	20	1		15	4	1	
416	340	55	0	0	17	1	1	20	1		18	1	1	
417	340	55	0	1	18	1	1	20	1		17	-1	1	
418	341	68	0	0	16	1	1	20	1		18	2	1	
419	342	74	1	0	17	0	0	20	1	20	26	9	0	2
420	343	57	0	1	19	1	0	20	1	36	31	12	0	0
421	344	80	0	0	12	0	1	20	1		15	3	1	
422	345	63	1	1	12	3	1	20	1		16	4	1	
423	346	29	1	0	13	3	0	20	1	16	30	17	0	0
424	347	70	0	0	10	0	1	20	1		15	5	1	
425	348	75	1	0	11	0	1	20	1		11	0	1	
426	349	42	0	0	8	1	1	20	1		13	5	1	
427	349	42	0	1	8	1	1	20	1		10	2	1	
428	350	67	0	1	13	1	0	20	1	31	22	9	0	2
429	351	73	1	0	14	0	1	20	1		17	3	1	
430	352	60	1	1	13	1	1	20	1		19	6	0	
431	353	70	1	0	14	2	1	20	1		15	1	1	
432	353	70	1	1	15	2	1	20	1		16	1	1	
433	354	64	1	0	14	1	1	20	1		18	4	1	
434	354	64	1	1	15	1	1	20	1		19	4	1	
435	355	72	0	0	15	1	0	20	1	41	24	9	0	2
436	356	80	1	0	16	0	1	20	1		19	3	1	
437	357	54	0	0	17	1	0	20	1	28	29	12	0	0
438	358	42	1	0	15	1	1	20	1		20	5	1	
439	358	42	1	1	19	1	1	20	1		21	2	1	
440	359	55	0	1	11	1	1	20	1		13	2	1	
441	360	53	1	0	18	1	0	20	1	85	30	12	0	0
442	361	77	1	0	14	0	1	20	1		19	5	1	
443	362	85	1	0	12	3	1	20	1		14	2	1	
444	363	71	0	1	13	0	1	20	1		21	8	0	
445	364	67	0	1	14	0	1	20	1		15	1	1	
446	365	62	1	0	18	0	1	20	1		19	1	1	
447	366	53	0	0	15	0	1	20	1		17	2	1	
448	367	60	1	1	13	0	1	20	1		14	1	1	
449	368	78	0	0	15	0	1	20	1		17	2	1	
450	369	82	0	0	10	0	1	20	1		15	5	1	
451	370	44	1	0	17	3	1	20	1		20	3	1	
452	371	73	0	0	10	0	1	20	1		17	7	0	
453	372	69	1	1	15	1	1	20	1		19	4	1	
454	373	65	1	1	14	0	0	20	1	131	23	9	0	0
455	374	73	0	0	16	1	0	20	1	92	24	8	0	2
456	375	73	1	0	17	1	1	20	1		18	1	1	
457	375	73	1	1	16	1	1	20	1		18	2	1	
458	376	70	1	1	14	0	1	20	1		16	2	1	
459	377	35	0	0	16	1	0	20	1	60	24	8	0	0
460	377	35	0	1	12	1	0	20	1	58	23	11	0	0
461	378	87	0	1	11	3	1	20	1		12	1	1	
462	379	69	0	1	14	1	1	20	1		19	5	1	
463	380	77	1	0	15	2	1	20	1		17	2	1	
464	380	77	1	1	13	2	1	20	1		17	4	1	
465	381	58	1	0	16	0	1	20	1		17	1	1	
466	382	78	1	0	9	0	1	20	1		15	6	0	
467	383	71	1	0	10	2	1	20	1		17	7	0	
468	384	79	0	0	11	0	1	20	1		14	3	1	
469	385	71	1	1	17	1	1	20	1		17	0	1	
470	386	39	1	1	18	3	1	20	1		18	0	1	
471	387	45	1	0	13	2	1	20	1		18	5	1	
472	388	56	1	0	18	1	0	20	1	42	24	6	0	0
473	388	56	1	1	18	1	0	20	1	44	29	11	0	0
474	389	75	0	1	14	0	1	20	1		18	4	1	
475	390	76	1	0	14	0	1	20	1		18	4	1	
476	391	54	1	0	19	0	1	20	1		19	0	1	
477	392	71	0	1	15	0	1	20	1		19	4	1	
478	393	57	0	1	14	0	1	20	1		16	2	1	
479	394	43	0	1	13	0	1	20	1		12	-1	1	
480	395	57	1	0	13	0	1	20	1		20	7	0	
481	396	41	1	0	12	1	1	20	1		15	3	1	
482	397	65	0	1	11	0	1	20	1		15	4	1	
483	398	64	1	1	13	0	1	20	1		15	2	1	
484	399	61	1	0	16	1	1	20	1		20	4	1	
485	399	61	1	1	15	1	1	20	1		21	6	0	
486	400	64	0	1	16	1	1	20	1		17	1	1	
487	401	67	0	1	13	0	1	20	1		17	4	1	
488	402	18	1	1	19	0	1	20	1		21	2	1	
489	403	72	1	1	11	1	1	20	1		13	2	1	
490	404	67	1	0	11	3	1	20	1		11	0	1	
491	404	67	1	1	10	3	1	20	1		14	4	1	
492	405	87	1	1	14	1	1	20	1		14	0	1	
493	406	76	0	1	10	2	1	20	1		18	8	0	
494	407	41	1	1	19	3	1	20	1		19	0	1	
495	408	66	1	1	15	1	1	20	1		18	3	1	
496	409	52	0	0	18	1	1	20	1		21	3	1	
497	410	77	0	1	16	1	1	20	1		21	5	1	
498	411	63	1	0	20	0	1	20	1		20	0	1	
499	412	78	0	1	9	0	1	20	1		15	6	0	
500	413	74	1	1	12	1	1	20	1		14	2	1	
501	414	56	1	1	14	3	0	20	1	29	25	11	0	2
502	415	65	1	0	19	1	1	20	1		18	-1	1	
503	416	75	1	0	16	1	1	20	1		17	1	1	
504	416	75	1	1	18	1	1	20	1		18	0	1	
505	417	72	0	0	14	0	1	20	1		18	4	1	
506	418	57	1	0	19	1	0	20	1	29	28	9	0	0
507	419	57	1	0	17	1	0	20	1	34	42	25	0	2
508	419	57	1	1	18	1	0	20	1	34	42	24	0	0
509	420	66	0	1	10	1	1	20	1		15	5	1	
510	421	87	0	1	9	3	1	20	1		12	3	1	
511	422	67	1	1	13	1	1	20	1		19	6	0	
512	423	61	1	1	16	2	1	20	1		20	4	1	
513	424	44	0	0	13	1	1	20	1		14	1	1	
514	424	44	0	1	14	1	1	20	1		16	2	1	
515	425	65	0	1	17	1	1	20	1		21	4	1	
516	426	55	0	1	18	2	0	20	1	85	29	11	0	0
517	427	88	0	0	9	1	1	20	1		10	1	1	
518	428	57	0	1	15	3	1	20	1		21	6	0	
519	429	40	0	0	16	1	1	20	1		18	2	1	
520	430	71	1	0	17	0	1	20	1		17	0	1	
521	430	71	1	1	13	1	1	20	1		16	3	1	
522	431	64	1	0	16	1	1	20	1		20	4	1	
523	431	64	1	1	18	1	1	20	1		20	2	1	
524	432	33	1	1	13	3	1	20	1		14	1	1	
525	433	78	1	1	11	3	1	20	1		18	7	0	
526	434	81	1	1	12	1	1	20	1		18	6	0	
527	435	79	0	0	13	1	1	20	1		19	6	0	
528	436	60	0	1	17	1	1	20	1		21	4	1	
529	437	75	0	0	15	1	1	20	1		18	3	1	
530	438	63	0	1	13	1	1	20	1		14	1	1	
531	439	63	1	0	16	1	1	20	1		18	2	1	
532	440	57	0	0	13	1	1	20	1		17	4	1	
533	440	57	0	1	13	1	1	20	1		16	3	1	
534	441	77	1	0	18	2	1	20	1		19	1	1	
535	441	77	1	1	17	2	1	20	1		19	2	1	
536	442	61	0	1	12	1	1	20	1		19	7	0	
537	443	78	1	0	13	2	1	20	1		17	4	1	
538	444	77	1	0	16	0	1	20	1		18	2	1	
539	445	76	0	0	17	1	1	20	1		21	4	1	
540	445	76	0	1	14	1	1	20	1		21	7	0	
541	446	64	1	0	10	1	1	20	1		18	8	0	
542	447	51	1	0	15	0	1	20	1		19	4	1	
543	448	65	1	0	15	2	1	20	1		19	4	1	
544	448	65	1	1	16	2	1	20	1		18	2	1	
545	449	74	1	0	10	1	1	20	1		12	2	1	
546	450	51	0	1	18	0	0	20	1	123	23	5	1	2
547	451	57	1	0	11	2	0	20	1	13	32	21	0	0
548	451	57	1	1	12	2	0	20	1	13	28	16	0	2
549	452	44	0	0	12	2	0	20	1	41	24	12	0	0
550	452	44	0	1	14	2	0	20	1	41	23	9	0	0
551	453	32	0	1	12	1	1	20	1		16	4	1	
552	454	63	0	1	12	1	1	20	1		15	3	1	
553	455	70	1	1	13	1	1	20	1		18	5	1	
554	456	62	1	1	9	1	1	20	1		20	11	0	
555	457	41	1	1	18	1	1	20	1		21	3	1	
556	458	67	0	1	19	1	1	20	1		20	1	1	
557	459	62	1	0	12	2	1	20	1		13	1	1	
558	460	75	0	1	18	0	1	20	1		18	0	1	
559	461	60	1	0	17	1	0	20	1	115	23	6	0	0
560	461	60	1	1	19	1	0	20	1	127	22	3	1	0
561	462	65	0	0	15	1	1	20	1		18	3	1	
562	463	68	0	0	15	1	1	20	1		18	3	1	
563	463	68	0	1	14	1	1	20	1		21	7	0	
564	464	52	0	1	13	1	1	20	1		18	5	1	
565	465	66	0	1	19	2	0	20	1	92	24	5	1	0
566	466	79	1	1	14	1	1	20	1		19	5	1	
567	467	85	0	0	10	0	1	20	1		18	8	0	
568	468	51	0	1	14	1	1	20	1		21	7	0	
569	469	63	1	0	18	1	1	20	1		19	1	1	
570	470	94	1	0	15	0	1	20	1		19	4	1	
571	471	75	1	0	18	0	0	20	1	27	32	14	0	0
572	472	67	0	0	20	1	1	20	1		20	0	1	
573	472	67	0	1	19	1	1	20	1		19	0	1	
574	473	63	0	1	18	1	0	20	1	92	25	7	0	2
575	474	60	1	0	8	2	1	20	1		11	3	1	
576	475	49	0	0	17	1	1	20	1		21	4	1	
577	475	49	0	1	19	1	1	20	1		20	1	1	
578	476	37	0	0	12	1	1	20	1		18	6	0	
579	476	37	0	1	13	1	1	20	1		21	8	0	
580	477	51	0	1	21	1	0	20	1	181	23	2	1	2
581	478	65	1	0	16	2	1	20	1		19	3	1	
582	478	65	1	1	17	2	1	20	1		20	3	1	
583	479	74	1	0	10	2	1	20	1		19	9	0	
584	480	74	1	1	12	1	1	20	1		13	1	1	
585	481	61	1	0	19	1	0	20	1	145	22	3	1	2
586	481	61	1	1	19	1	0	20	1	132	22	3	1	2
587	482	59	0	1	16	2	1	20	1		20	4	1	
588	483	64	0	0	16	1	1	20	1		17	1	1	
589	483	64	0	1	15	1	1	20	1		19	4	1	
590	484	70	1	1	16	1	1	20	1		17	1	1	
591	485	77	0	1	14	3	1	20	1		18	4	1	
592	486	52	0	0	12	1	1	20	1		14	2	1	
593	486	52	0	1	11	1	1	20	1		13	2	1	
594	487	83	0	0	19	1	1	20	1		21	2	1	
595	487	83	0	1	19	1	1	20	1		19	0	1	
596	488	57	0	1	13	0	1	20	1		15	2	1	
597	489	69	0	0	16	1	1	20	1		18	2	1	
598	489	69	0	1	15	1	1	20	1		16	1	1	
599	490	65	1	1	16	1	1	20	1		17	1	1	
600	491	45	0	0	16	1	1	20	1		17	1	1	
601	491	45	0	1	10	1	1	20	1		17	7	0	
602	492	38	0	0	19	1	1	20	1		20	1	1	
603	492	38	0	1	18	1	1	20	1		19	1	1	
604	493	66	0	0	16	0	1	20	1		19	3	1	
605	494	64	0	0	13	1	1	20	1		17	4	1	
606	495	54	0	0	15	1	1	20	1		19	4	1	
607	496	60	1	0	15	1	1	20	1		21	6	0	
608	496	60	1	1	14	1	1	20	1		18	4	1	
609	497	83	0	0	10	1	1	20	1		19	9	0	
610	498	83	1	1	17	1	1	20	1		19	2	1	
611	499	73	0	1	13	2	1	20	1		15	2	1	
612	500	49	0	0	11	1	1	20	1		14	3	1	
613	500	49	0	1	10	1	1	20	1		15	5	1	
614	501	41	0	0	17	1	1	20	1		20	3	1	
615	501	41	0	1	15	1	1	20	1		21	6	0	
616	502	47	0	1	17	0	0	20	1	63	34	17	0	0
617	503	66	0	0	13	1	1	20	1		16	3	1	
618	504	61	1	1	14	1	1	20	1		16	2	1	
619	505	75	0	0	16	0	1	20	1		20	4	1	
620	506	32	1	1	17	2	1	20	1		20	3	1	
621	507	36	1	0	17	2	1	20	1		20	3	1	
622	507	36	1	1	15	2	1	20	1		21	6	0	
623	508	41	0	1	18	1	1	20	1		18	0	1	
624	509	46	1	0	14	1	1	20	1		18	4	1	
625	509	46	1	1	14	1	1	20	1		18	4	1	
626	510	64	1	1	15	2	1	20	1		18.3	3	1	
627	511	72	0	1	17	0	1	20	1		17	0	1	
628	512	76	1	1	11	2	1	20	1		15	4	1	
629	513	63	0	0	11	2	1	20	1		11	0	1	
630	513	63	0	1	9	2	1	20	1		11	2	1	
631	514	72	1	0	11	2	1	20	1		11	0	1	
632	514	72	1	1	9	2	1	20	1		10	1	1	
633	515	29	0	1	12	2	1	20	1		18	6	0	
634	516	62	0	0	12	2	1	20	1		15	3	1	
635	517	66	1	0	15	2	1	20	1		18	4	1	
636	518	47	0	0	16	1	1	20	1		18	3	1	
637	518	47	0	1	14	1	1	20	1		17	3	1	
638	519	27	0	0	12	0	1	20	1		17	5	1	
639	519	27	0	1	11	0	1	20	1		17	6	0	
640	520	69	1	1	19	1	1	20	1		18.7	0	1	
641	521	81	0	1	12	2	1	20	1		17	5	1	
642	522	85	0	1	11	0	1	20	1		12.3	1	1	
643	523	73	1	1	12.5	2	1	20	1		16	4	1	
644	524	68	1	0	13	2	1	20	1		17.5	5	1	
645	525	80	1	1	9	2	1	20	1		12	3	1	
646	526	64	1	0	13	2	0	20	1	162	22	9	0	0
647	527	74	0	0	8	2	1	20	1		14	6	0	
648	527	74	0	1	10	2	1	20	1		20	10	0	
649	528	49	0	1	20.1	2	0	20	1	60	29	9	0	0
650	529	62	0	1	11	1	1	20	1		11	0	1	
651	530	78	0	1	14.5	3	1	20	1		16	2	1	
652	531	59	1	1	11.5	3	1	20	1		14	3	1	
653	532	68	0	1	8	0	1	20	1		11.5	4	1	
654	533	60	0	0	10	2	1	20	1		10	0	1	
655	534	63	0	0	11	1	1	20	1		15	4	1	
656	534	63	0	1	10.5	1	1	20	1		15	5	1	
657	535	66	1	0	12	2	1	20	1		12	0	1	
658	536	56	0	0	16	2	1	20	1		19.7	4	1	
659	537	82	0	1	17.5	0	1	20	1		18	1	1	
660	538	75	0	1	16	0	1	20	1		16	0	1	
661	539	64	0	1	10	2	1	20	1		11	1	1	
662	540	48	0	1	10	2	1	20	1		16.7	7	0	
663	541	42	1	0	16	2	1	20	1		16	0	1	
664	542	78	1	0	12.5	0	1	20	1		15	3	1	
665	543	81	0	0	13.3	3	1	20	1		17	4	1	
666	544	62	0	1	18	1	1	20	1		18	0	1	
667	545	62	0	1	11	2	1	20	1		13	2	1	
668	546	55	1	0	9	2	1	20	1		13	4	1	
669	547	60	0	0	17	0	1	20	1		19.5	3	1	
670	547	60	0	1	16	0	1	20	1		19	3	1	
671	548	65	1	0	11	2	1	20	1		12	1	1	
672	548	73	1	1	11	2	1	20	1		12	1	1	
673	549	60	1	0	11	0	1	20	1		15.5	5	1	
674	550	61	0	0	11	1	1	20	1		14	3	1	
675	551	34	0	0	17	3	0	20	1	90	22.7	6	0	0
676	552	61	1	1	13	3	0	20	1	34	23.7	11	0	0
677	553	69	1	0	10	2	1	20	1		14	4	1	
678	553	69	1	1	11	2	1	20	1		16	5	1	
679	554	76	1	1	13	2	1	20	1		13	0	1	
680	555	60	0	0	12.5	2	0	20	1	106	31.5	19	0	0
681	556	57	0	0	11	1	1	20	1		16.5	6	0	
682	556	57	0	1	9.5	1	1	20	1		14	5	1	
683	557	73	1	0	15	0	1	20	1		18	3	1	
684	558	42	1	1	14	2	0	20	1	57	25.3	11	0	0
685	559	61	0	0	16.3	1	1	20	1		19	3	1	
686	560	58	0	0	12	0	1	20	1		16.5	5	1	
687	561	75	1	1	11	0	1	20	1		13	2	1	
688	562	71	1	1	15	2	1	20	1		16	1	1	
689	563	65	1	1	14	1	1	20	1		19.7	6	0	
690	564	64	0	1	9.5	0	1	20	1		14	5	1	
691	565	54	0	0	14	1	1	20	1		14.7	1	1	
692	566	23	1	0	18	3	1	20	1		19	1	1	
693	567	56	0	1	12.7	2	1	20	1		18	5	1	
694	568	64	0	0	14	3	1	20	1		20.7	7	0	
695	569	64	0	0	14.5	2	1	20	1		17	3	1	
696	569	64	0	1	13	2	1	20	1		16	3	1	
697	570	60	1	1	11	1	1	20	1		11	0	1	
698	571	48	0	1	11	1	1	20	1		12	1	1	
699	572	54	0	0	16.5	1	1	20	1		21	5	1	
700	573	56	1	0	11.5	2	1	20	1		17	6	0	
701	573	56	1	1	12	2	1	20	1		17	5	1	
702	574	60	0	1	3	2	1	20	1		9.7	7	0	
703	575	66	0	0	12	1	1	20	1		14	2	1	
704	576	40	1	0	18	2	0	20	1	7	23	5	1	0
705	576	40	1	1	18	2	0	20	1	7	26	8	0	0
706	577	53	1	0	13	2	1	20	1		13	0	1	
707	577	53	1	1	11	2	1	20	1		12.5	2	1	
708	578	77	0	1	9	1	1	20	1		12	3	1	
709	579	49	1	1	8	1	1	20	1		19	11	0	
710	580	74	0	0	17	1	1	20	1		20	3	1	
711	581	63	1	0	13	2	1	20	1		15.3	2	1	
712	582	69	1	0	17.5	1	1	20	1		19	2	1	
713	582	69	1	1	14	1	0	20	1	112	22.2	8	0	2
714	583	64	1	0	16.5	1	0	20	1	64	25.5	9	0	0
715	584	43	0	1	19	2	1	20	1		20	1	1	
716	585	70	1	0	19	2	1	20	1		19	0	1	
717	586	73	1	0	10	3	1	20	1		12	2	1	
718	587	80	1	1	13	2	1	20	1		13	0	1	
719	588	67	1	1	14	2	1	20	1		14	0	1	
720	589	58	1	1	15.3	2	1	20	1		19.5	4	1	
721	590	72	1	0	17	2	1	20	1		18.3	1	1	
722	591	68	0	1	15	3	1	20	1		17.5	3	1	
723	592	50	0	0	16	3	1	20	1		19	3	1	
724	593	66	0	0	11	2	1	20	1		15	4	1	
725	593	66	0	1	10	2	1	20	1		13	3	1	
726	594	77	0	0	16	2	1	20	1		20.7	5	1	
727	594	77	0	1	16	2	1	20	1		18	2	1	
728	595	71	1	1	15	2	1	20	1		18	3	1	
729	596	79	0	1	11	2	1	20	1		13	2	1	
730	597	48	1	0	5	2	1	20	1		12	7	0	
731	597	48	1	1	6	2	1	20	1		11	5	1	
732	598	56	1	1	17	2	0	20	1	123	27.7	11	0	0
733	599	81	1	1	10	0	1	20	1		13	3	1	
734	600	41	1	0	13.5	3	1	20	1		13.5	0	1	
735	601	81	0	1	11	3	1	20	1		14	3	1	
736	602	76	0	1	9	0	1	20	1		12	3	1	
737	603	67	1	0	14	1	1	20	1		17	3	1	
738	603	67	1	1	14	1	1	20	1		17	3	1	
739	604	71	0	1	12	1	1	20	1		13	1	1	
740	605	78	1	1	15	2	1	20	1		15	0	1	
741	606	37	1	1	20	1	0	20	1	182	24.7	5	1	0
742	607	69	1	1	8	0	1	20	1		11	3	1	
743	608	64	0	0	12	1	1	20	1		14	2	1	
744	608	64	0	1	10.7	1	1	20	1		14	3	1	
745	609	47	0	0	12	1	1	20	1		13	1	1	
746	610	90	1	1	7.5	2	1	20	1		10	3	1	
747	611	44	1	0	14	1	1	20	1		18	4	1	
748	612	70	1	0	13	2	1	20	1		19	6	0	
749	613	58	1	0	11	2	1	20	1		16	5	1	
750	614	80	0	0	16.5	3	1	20	1		15	-2	1	
751	614	80	0	1	11	3	1	20	1		18	7	0	
752	615	68	1	1	18.3	1	1	20	1		19	1	1	
753	616	67	0	1	17	0	1	20	1		17	0	1	
754	617	78	0	1	18	2	1	20	1		18	0	1	
755	618	42	1	0	18.5	3	0	20	1	47	23.7	5	1	0
756	618	42	1	1	19	3	0	20	1	47	23.3	4	1	0
757	619	76	1	0	12	3	1	20	1		15.3	3	1	
758	620	45	0	0	11	2	1	20	1		11.7	1	1	
759	621	62	0	1	18.5	1	1	20	1		18.5	0	1	
760	622	62	1	0	18.3	3	1	20	1		18.5	0	1	
761	623	45	0	0	18	1	1	20	1		18.3	0	1	
762	624	67	1	1	15	2	1	20	1		19	4	1	
763	625	65	1	0	12	2	1	20	1		15	3	1	
764	625	65	1	1	9.7	2	1	20	1		15	5	1	
765	626	62	0	0	6.5	0	1	20	1		9	3	1	
766	627	62	0	1	10.3	1	1	20	1		14.5	4	1	
767	628	66	1	0	12.5	2	1	20	1		14	2	1	
768	628	66	1	1	13	2	1	20	1		13.5	1	1	
769	629	73	1	0	13	2	1	20	1		16	3	1	
770	629	73	1	1	12	2	1	20	1		16	4	1	
771	630	72	0	0	12.5	2	1	20	1		21	9	0	
772	630	72	0	1	11	2	1	20	1		17.7	7	0	
773	631	63	0	0	11	1	1	20	1		15	4	1	
774	631	63	0	1	12	1	1	20	1		12	0	1	
775	632	44	0	0	10	1	1	20	1		12	2	1	
776	632	44	0	1	10	1	1	20	1		11	1	1	
777	633	46	1	1	14	2	1	20	1		18	4	1	
778	634	81	1	1	7	2	1	20	1		9	2	1	
779	635	54	1	1	17.5	1	1	20	1		19	2	1	
780	636	64	1	0	13	2	1	20	1		18.7	6	0	
781	637	56	0	0	11	1	1	20	1		13	2	1	
782	637	56	0	1	11	1	1	20	1		12	1	1	
783	638	61	1	1	15	2	1	20	1		18	3	1	
784	639	48	0	0	11.3	2	1	20	1		16	5	1	
785	640	63	1	1	11.3	3	1	20	1		12	1	1	
786	641	54	1	1	11	2	1	20	1		13	2	1	
787	642	61	1	0	19.3	3	0	20	1	50	31.7	12	0	0
788	643	37	1	1	16	3	1	20	1		17	1	1	
789	644	76	1	0	15	0	1	20	1		13	-2	1	
790	645	88	1	1	16	0	1	20	1		20	4	1	
791	646	64	1	1	14	0	1	20	1		18	4	1	
792	647	87	1	1	13	3	1	20	1		15	2	1	
793	648	78	1	0	14	0	1	20	1		15	1	1	
794	649	85	0	1	17	2	1	20	1		16	-1	1	
795	650	81	0	1	14	3	1	20	1		14	0	1	
796	651	69	1	1	21	1	0	20	1	180	24	3	1	2
797	652	69	1	0	16	0	0	20	1	120	22	6	0	2
798	653	72	1	1	18	0	0	20	1	390	22	4	1	2
799	654	59	0	1	14	0	1	20	1		16	2	1	
800	655	69	0	0	12	1	1	20	1		20	8	0	
801	656	73	0	0	20	0	1	20	1		20	0	1	
802	657	84	1	1	17	3	1	20	1		11	-6	1	
803	658	69	1	0	14	1	1	20	1		20	6	0	
804	659	64	0	1	16	0	1	20	1		20	4	1	
805	660	66	0	0	18	1	0	20	1	270	26	8	0	2
806	661	64	0	1	18	1	0	20	1	18	31	13	0	0
807	662	69	1	0	13	0	1	20	1		16	3	1	
808	663	84	0	1	13	0	1	20	1		16	3	1	
809	664	81	0	0	20	0	1	20	1		14	-6	1	
810	665	63	1	0	15	0	1	20	1		20	5	1	
811	666	74	0	0	15	0	1	20	1		19	4	1	
812	667	63	1	0	13	3	1	20	1		16	3	1	
813	668	67	1	1	13	3	1	20	1		17	4	1	

814	669	67	0	0	16	3	1	20	1		15	-1	1	
815	670	66	0	1	12	3	1	20	1		12	0	1	
816	671	72	1	0	16	1	1	20	1		15	-1	1	
817	672	86	1	1	11	1	1	20	1		9	-2	1	
818	673	82	0	1	18	3	0	20	1	120	28	10	0	2
819	674	81	1	0	6	3	1	20	1		12	6	0	
820	675	75	1	1	13	3	1	20	1		13	0	1	
821	676	56	0	0	14	1	1	20	1		17	3	1	
822	677	62	0	1	10	2	1	20	1		17	7	0	
823	678	63	1	0	14	3	1	20	1		17	3	1	
824	679	84	0	1	8	3	1	20	1		7	-1	1	
825	680	77	0	1	14	3	1	20	1		14	0	1	
826	681	80	0	1	7	3	1	20	1		9	2	1	
827	682	59	1	0	15	3	1	20	1		18	3	1	
828	683	74	1	1	17	3	1	20	1		17	0	1	
829	684	66	1	1	7	0	1	20	1		9	2	1	
830	685	74	1	0	14	1	1	20	1		16	2	1	
831	686	76	0	0	7	1	1	20	1		12	5	1	
832	687	85	0	1	11	0	1	20	1		13	2	1	
833	688	69	0	1	14	1	0	20	1	48	25	11	0	0
834	689	70	0	0	8	1	1	20	1		8	0	1	
835	690	42	0	0	13	1	1	20	1		17	4	1	
836	691	79	1	0	12	0	1	20	1		15	3	1	
837	692	66	0	0	16	1	1	20	1		18	2	1	
838	693	81	0	1	7	1	1	20	1		10	3	1	
839	694	63	0	0	11	1	1	20	1		10	-1	1	
840	695	74	1	1	17	1	1	20	1		15	-2	1	
841	696	64	1	0	15	1	1	20	1		17	2	1	
842	697	70	0	1	10	1	1	20	1		13	3	1	
843	698	77	0	1	8	1	1	20	1		7	-1	1	
844	699	64	1	1	16	0	1	20	1		19	3	1	
845	700	64	0	1	16	1	1	20	1		15	-1	1	
846	701	66	0	0	11	1	1	20	1		10	-1	1	
847	702	63	0	1	13	1	1	20	1		18	5	1	
848	703	75	0	0	10	1	1	20	1		14	4	1	
849	704	58	0	0	14	2	1	20	1		15	1	1	
850	705	56	0	1	13	1	1	20	1		17	4	1	
851	706	78	1	1	14	1	1	20	1		16	2	1	
852	707	75	1	1	14	1	1	20	1		20	6	0	
853	708	66	0	0	12	0	0	20	1	84	34	22	0	0
854	709	75	0	0	13	0	1	20	1		18	5	1	
855	710	77	1	0	7	1	1	20	1		10	3	1	
856	711	65	0	1	12	1	1	20	1		16	4	1	
857	712	55	0	0	15	0	1	20	1		18	3	1	
858	713	61	0	0	10	1	1	20	1		15	5	1	
859	714	66	1	1	12	2	1	20	1		14	2	1	
860	715	68	0	0	14	1	1	20	1		16	2	1	
861	716	44	1	1	11	2	1	20	1		16	5	1	
862	717	70	0	1	14	1	1	20	1		11	-3	1	
863	718	57	0	1	14	0	1	20	1		21	7	0	
864	719	79	0	1	8	1	1	20	1		11	3	1	
865	720	67	1	0	9	1	1	20	1		13	4	1	
866	721	49	0	1	15	1	1	20	1		15	0	1	
867	722	33	0	0	14	1	1	20	1		19	5	1	
868	723	83	1	1	9	1	1	20	1		12	3	1	
869	724	57	0	0	15	1	0	20	1	64	22	7	0	2
870	725	52	1	0	17	1	1	20	1		20	3	1	
871	726	78	0	1	14	1	1	20	1		18	4	1	
872	727	52	1	0	15	1	1	20	1		16	1	1	
873	728	63	1	0	9	1	1	20	1		13	4	1	
874	729	65	0	1	17	1	1	20	1		20	3	1	
875	730	60	0	1	14	1	1	20	1		18	4	1	
876	731	46	0	1	13	1	1	20	1		16	3	1	
877	732	64	1	1	16	1	1	20	1		17	1	1	
878	733	75	0	1	14	1	1	20	1		14	0	1	
879	734	41	0	1	15	1	1	20	1		17	2	1	
880	735	51	1	1	16	1	1	20	1		20	4	1	
881	736	70	0	1	8	1	1	20	1		12	4	1	
882	737	46	0	1	20	1	0	20	1	10	23	3	1	0
883	738	65	0	1	16	1	1	20	1		17	1	1	
884	739	57	0	1	11	1	1	20	1		14	3	1	
885	740	68	0	0	15	1	1	20	1		21	6	0	
886	741	71	0	0	12	1	1	20	1		13	1	1	
887	742	73	0	1	13	1	1	20	1		15	2	1	
888	743	62	1	1	21	1	1	20	1		17	-4	1	
889	744	69	0	0	16	1	1	20	1		21	5	1	
890	745	39	0	0	14	1	0	20	1	90	22	8	0	2
891	746	63	0	0	10	1	1	20	1		12	2	1	
892	747	40	0	1	11	1	1	20	1		18	7	0	
893	748	77	0	1	7	1	1	20	1		8	1	1	
894	749	67	0	0	8	1	1	20	1		10	2	1	
895	750	68	1	0	9	1	1	20	1		8	-1	1	
896	751	52	0	1	12	1	1	20	1		16	4	1	
897	752	74	0	1	14	1	1	20	1		18	4	1	
898	753	68	1	0	14	1	1	20	1		16	2	1	
899	754	59	0	0	15	1	0	20	1	40	28	13	0	0
900	755	52	0	0	15	1	1	20	1		20	5	1	
901	756	67	0	0	13	1	1	20	1		17	4	1	
902	757	63	0	0	12	1	0	20	1	16	25	13	0	0
903	758	68	1	0	17	0	1	20	1		19	2	1	
904	759	46	0	1	15	1	1	20	1		18	3	1	
905	760	60	1	0	13	1	1	20	1		18	5	1	
906	761	66	1	1	14	1	1	20	1		16	2	1	
907	762	64	1	0	17	1	1	20	1		18	1	1	
908	763	68	1	0	11	0	1	20	1		17	6	0	
909	764	73	1	0	17	1	1	20	1		17	0	1	
910	765	59	0	0	15	1	1	20	1		20	5	1	
911	766	72	0	1	14	1	1	20	1		16	2	1	
912	767	74	0	1	11	1	1	20	1		11	0	1	
913	768	46	0	1	9	1	1	20	1		17	8	0	
914	769	57	1	1	12	1	1	20	1		14	2	1	
915	770	60	1	1	15	0	1	20	1		19	4	1	
916	771	24	0	0	13	2	0	20	1	20	29	16	0	0
917	772	41	0	1	18	2	0	20	1	7	22	4	1	2
918	773	61	1	0	12	1	1	20	1		15	3	1	
919	774	74	1	1	9	0	1	20	1		12	3	1	
920	775	47	1	0	12	0	1	20	1		16	4	1	
921	776	68	1	0	13	0	1	20	1		13	0	1	
922	777	90	0	1	7	1	1	20	1		15	8	0	
923	778	57	1	1	15	1	1	20	1		19	4	1	
924	779	62	1	1	10	0	1	20	1		10	0	1	
925	780	39	1	1	12	3	1	20	1		16	4	1	
926	781	76	1	1	18	1	1	20	1		18	0	1	
927	782	62	1	1	14	1	1	20	1		18	4	1	
928	783	66	1	0	13	1	1	20	1		16	3	1	
929	784	72	0	1	14	1	1	20	1		18	4	1	
930	785	61	0	1	11	1	1	20	1		15	4	1	
931	786	66	1	0	8	0	1	20	1		13	5	1	
932	787	57	0	0	17	1	0	20	1	138	22	5	1	0
933	788	57	0	0	15	1	1	20	1		18	3	1	
934	789	81	0	0	17	1	1	20	1		15	-2	1	
935	790	58	0	0	8	1	1	20	1		18	10	0	
936	791	37	0	1	11	1	1	20	1		21	10	0	
937	792	50	0	0	17	1	1	20	1		21	4	1	
938	793	67	0	0	13	1	1	20	1		13	0	1	
939	794	83	0	1	12	1	1	20	1		12	0	1	
940	795	82	0	1	14	1	1	20	1		14	0	1	
941	796	75	1	0	12	2	1	20	1		17	5	1	
942	797	75	0	0	10	0	1	20	1		12	2	1	
943	798	55	0	0	11	2	1	20	1		12	1	1	
944	799	76	0	0	10	1	1	20	1		17	7	0	
945	800	74	1	0	10	1	1	20	1		13	3	1	
946	801	76	1	0	19	1	0	20	1	90	23	4	1	2
947	802	72	1	1	10	1	1	20	1		13	3	1	
948	803	53	0	0	15	1	1	20	1		14	-1	1	
949	804	72	1	1	10	2	1	20	1		17	7	0	
950	805	67	0	0	9	1	1	20	1		14	5	1	
951	806	49	1	0	17	1	1	20	1		20	3	1	
952	807	62	1	1	16	3	1	20	1		10	-6	1	
953	808	70	0	0	9	1	1	20	1		16	7	0	
954	809	44	0	1	11	1	1	20	1		15	4	1	
955	810	61	1	0	16	2	0	20	1	145	22	6	0	2
956	810	61	1	1	16	2	0	20	1	37	22	6	0	2
957	811	62	1	1	14	0	1	20	1		21	7	0	
958	812	42	0	1	15	2	0	20	1	77	24	9	0	0
959	813	43	1	1	17	0	0	20	1	47	22	5	1	2
960	814	72	0	0	13	0	1	20	1		19	6	0	
961	815	58	1	0	16	0	0	80	4	149	22	6	0	2
962	816	79	1	1	18	1	0	20	1	35	22	4	1	2
963	817	36	0	0	21	1	0	20	1	113	23	2	1	2
964	818	60	1	0	12	0	0	40	2	148	24	12	0	2
965	819	74	0	0	19	1	0	40	2	35	33	14	0	0
966	820	50	0	1	14	1	0	20	1	146	23	9	0	2
967	821	75	0	0	15	0	0	20	1	253	30	15	0	2
968	822	64	0	1	18	0	0	40	2	35	28	10	0	0
969	823	60	0	0	19	2	0	20	1	57	28	9	0	2
970	824	77	1	0	15	1	0	40	2	116	26	11	0	2
971	825	58	1	0	15	0	0	20	1	291	23	8	0	2
972	826	41	1	1	17	2	0	20	1	56	24	7	0	0
973	827	55	1	1	19	0	0	20	1	154	23	4	1	2
974	828	30	0	0	18	0	0	20	1	10	32	14	0	0
975	829	68	1	0	18	1	0	20	1	75	22	4	1	2
976	830	74	1	1	20	0	0	20	1	63	35	15	0	0
977	831	68	1	1	21	2	0	20	1	49	22	1	1	2
978	832	80	0	1	17	0	0	20	1	45	23	6	0	2
979	833	28	1	0	17	0	0	20	1	148	23	6	0	0
980	834	61	1	1	19	1	0	20	1	91	25	6	0	0
981	835	59	0	0	18	1	0	20	1	49	42	24	0	1
982	836	64	0	1	15	0	0	20	1	35	31	16	0	0
983	837	57	0	0	13	2	0	20	1	51	52	39	0	0
984	838	34	1	0	16	0	0	20	1	5	22	6	0	0
985	839	69	1	0	15	2	0	20	1	66	22	7	0	0
986	840	63	0	0	16	0	0	20	1	34	32	16	0	0
987	841	55	1	1	15	2	0	60	3	69	22	7	0	2
988	842	58	0	1	12	2	0	20	1	152	22	10	0	0
989	843	66	0	1	14	2	0	40	2	63	22	8	0	2
990	844	67	1	1	16	2	0	20	1	117	52	36	0	0
991	845	50	0	0	16	1	0	20	1	115	41	25	0	0
992	846	40	0	1	16	2	0	20	1	113	38	22	0	0
993	847	55	0	1	16	0	0	40	2	120	32	16	0	0
994	848	62	0	0	21	1	0	20	1	70	23	2	1	2
995	849	66	1	0	14	0	0	20	1	9	27	13	0	2
996	850	81	1	0	18	1	0	20	1	38	23	5	1	2
997	851	68	1	0	14	2	0	20	1	42	22	8	0	2
998	852	42	1	1	11	2	0	20	1	103	27	16	0	2
999	853	67	0	1	18	0	0	20	1	63	34	16	0	0
1000	854	61	1	1	18	0	0	20	1	56	24	6	0	2
1001	855	59	1	1	11	0	0	80	4	405	26	15	0	0
1002	856	58	0	0	12	1	0	20	1	84	23	11	0	0
1003	857	63	0	1	18	0	0	20	1	3	50	32	0	0
1004	858	79	0	0	15	1	0	20	1	95	32	17	0	0
1005	859	52	1	1	16	1	0	40	2	33	23	7	0	0
1006	860	79	0	0	18	0	0	40	2	49	24	6	0	2
1007	861	62	0	1	20	0	0	20	1	21	24	4	1	2
1008	862	72	0	0	16	0	0	40	2	21	22	6	0	2
1009	863	73	0	0	20	0	0	20	1	125	28	8	0	0
1010	864	70	0	1	21	0	0	20	1	7	22	1	1	2
1011	865	50	0	1	14	0	0	60	3	1	42	28	0	1
1012	866	55	1	1	16	1	0	20	1	280	24	8	0	2
1013	867	71	1	0	20	0	0	20	1	57	23	3	1	2
1014	868	56	0	0	14	0	0	60	3	49	22	8	0	2
1015	869	71	0	1	19	1	0	20	1	147	22	3	1	2
1016	870	22	0	0	18	2	0	20	1	95	22	4	1	2
1017	871	67	0	1	15	0	0	100	5	37	25	10	0	2
1018	872	57	1	1	15	0	0	60	3	56	27	12	0	0
1019	873	53	0	0	17	1	0	40	2	133	25	8	0	0
1020	874	54	1	0	14	0	0	20	1	118	25	11	0	2
1021	875	72	0	0	20	0	0	40	2	1	24	4	1	2
1022	876	68	1	0	15	0	0	20	1	8	25	10	0	0
1023	877	61	1	1	16	0	0	20	1	70	22	6	0	2
1024	878	29	0	0	20	2	0	20	1	67	31	11	0	0
1025	879	52	1	0	14	2	0	20	1	22	27	13	0	2
1026	880	64	0	0	14	0	0	40	2	1	26	12	0	2
1027	881	69	1	1	14	0	0	20	1	112	25	11	0	2
1028	882	59	1	0	19	0	0	20	1	35	22	3	1	2
1029	883	66	1	1	19	0	0	40	2	1	22	3	1	2
1030	884	42	0	0	19	1	0	40	2	111	25	6	0	2
1031	885	73	0	1	15	0	0	20	1	116	26	11	0	0
1032	886	64	1	1	18	1	0	20	1	71	26	8	0	0
1033	887	57	0	0	17	0	0	20	1	84	25	8	0	2
1034	888	57	0	0	17	1	0	20	1	75	23	6	0	2
1035	889	85	0	1	20	1	0	40	2	300	26	6	0	0
1036	890	66	0	1	21	1	0	20	1	49	23	2	1	2
1037	891	72	1	0	18	0	0	20	1	70	24	6	0	2
1038	892	67	0	0	17	1	0	20	1	128	22	5	1	2
1039	893	65	1	0	18	0	0	20	1	182	22	4	1	2
1040	894	52	0	0	17	1	0	20	1	48	24	7	0	2
1041	895	68	1	1	19	1	0	20	1	70	24	5	1	2
1042	896	36	1	0	18	2	0	40	2	98	22	4	1	0
1043	897	46	0	0	8	2	0	20	1	76	28	20	0	0
1044	898	79	1	1	16	1	0	20	1	32	22	6	0	2
1045	899	42	0	0	15	1	0	20	1	70	23	8	0	2
1046	900	61	0	1	17	0	0	20	1	14	72	55	0	0
1047	901	77	0	1	11	1	0	20	1	70	30	19	0	0
1048	902	58	0	0	13	0	0	20	1	40	24	11	0	2
1049	903	36	0	1	17	0	0	20	1	167	23	6	0	2
1050	904	64	1	1	17	0	0	20	1	68	25	8	0	0
1051	905	75	1	0	16	1	1	20	1		18	2	1	
1052	905	75	1	1	13	1	1	20	1		19	6	0	
1053	906	65	0	0	14	1	1	20	1		16	2	1	
1054	906	65	0	1	12	1	1	20	1		15	3	1	
1055	907	83	1	0	19	3	1	20	1		18	-1	1	
1056	908	61	1	1	12	1	1	20	1		16	4	1	
1057	909	68	0	0	16	1	1	20	1		19	3	1	
1058	910	54	0	1	15	1	1	20	1		20	5	1	
1059	911	51	0	0	16	1	1	20	1		18	2	1	
1060	912	72	0	1	14	0	1	20	1		15	1	1	
1061	913	48	1	1	19	1	1	20	1		20	1	1	
1062	914	44	1	1	17	0	1	20	1		21	4	1	
1063	915	59	0	0	14	3	1	20	1		19	5	1	
1064	916	57	1	0	15	2	1	20	1		17	2	1	
1065	917	75	0	1	15	1	1	20	1		17	2	1	
1066	917	75	0	0	15	1	1	20	1		19	4	1	
1067	918	49	1	1	16	2	1	20	1		20	4	1	
1068	919	60	1	1	13	1	1	20	1		19	6	0	
1069	920	89	0	1	11	0	1	20	1		17	6	0	
1070	921	48	0	0	16	2	1	20	1		17	1	1	
1071	922	62	0	1	15	0	1	20	1		18	3	1	
1072	923	58	0	1	13	0	1	20	1		17	4	1	
1073	924	61	1	0	12	0	1	20	1		15	3	1	
1074	925	72	0	1	11	0	1	20	1		15	4	1	
1075	926	53	1	0	12	2	1	20	1		18	6	0	
1076	926	53	1	1	13	2	1	20	1		16	3	1	
1077	927	61	0	0	19	1	1	20	1		14	-5	1	
1078	928	65	1	1	12	1	1	20	1		13	1	1	
1079	929	57	0	1	13	3	1	20	1		19	6	0	
1080	930	79	1	0	17	1	1	20	1		20	3	1	
1081	931	73	1	0	20	0	1	20	1		21	1	1	
1082	932	65	1	0	16	0	1	20	1		18	2	1	
1083	933	64	1	1	18	0	1	20	1		21	3	1	
1084	934	57	0	0	13	0	1	20	1		15	2	1	
1085	935	52	0	0	16	0	0	20	1	236	22	6	0	0
1086	936	62	0	0	16	1	1	20	1		19	3	1	
1087	936	62	0	1	17	1	1	20	1		17	0	1	
1088	937	76	1	0	11	0	1	20	1		15	4	1	
1089	938	80	1	1	12	0	1	20	1		15	3	1	
1090	939	43	0	1	15	0	1	20	1		19	4	1	
1091	940	62	0	1	16	1	1	20	1		19	3	1	
1092	941	73	0	1	18	0	1	20	1		18	0	1	
1093	942	70	0	0	13	0	1	20	1		18	5	1	
1094	943	68	1	0	10	1	1	20	1		16	6	0	
1095	943	68	1	1	11	1	1	20	1		17	6	0	
1096	944	51	1	1	15	1	1	20	1		19	4	1	
1097	945	79	1	0	11	0	1	20	1		16	5	1	
1098	946	69	0	1	17	1	1	20	1		21	4	1	
1099	947	65	0	1	12	0	1	20	1		18	6	0	
1100	948	67	0	1	11	0	1	20	1		16	5	1	
1101	949	69	1	1	13	0	1	20	1		14	1	1	
1102	950	48	0	0	17	1	1	20	1		20	3	1	
1103	951	58	0	1	10	1	1	20	1		10	0	1	
1104	952	70	1	1	17	0	1	20	1		17	0	1	
1105	953	74	0	1	14	1	1	20	1		14	0	1	
1106	954	65	0	1	13	1	1	20	1		19	6	0	
1107	955	75	0	0	12	0	1	20	1		19	7	0	
1108	956	57	1	1	18	0	1	20	1		20	2	1	
1109	957	74	1	0	13	0	1	20	1		16	3	1	
1110	958	38	1	1	19	2	1	20	1		19	0	1	
1111	959	53	0	1	12	0	1	20	1		20	8	0	
1112	960	74	1	0	11	2	1	20	1		18	7	0	
1113	961	68	0	0	8	0	1	20	1		11	3	1	
1114	962	80	1	0	17	1	1	20	1		19	2	1	
1115	963	76	0	1	14	0	1	20	1		14	0	1	
1116	964	56	1	1	13	0	1	20	1		19	6	0	
1117	965	68	1	1	19	0	1	20	1		19	0	1	
1118	966	54	1	1	20	0	1	20	1		19	-1	1	
1119	967	77	1	0	20	0	1	20	1		21	1	1	
1120	968	45	1	1	19	3	1	20	1		20	1	1	
1121	969	59	0	0	12	1	1	20	1		15	3	1	
1122	970	81	1	1	16	0	1	20	1		19	3	1	
1123	971	72	0	1	12	0	1	20	1		17	5	1	
1124	972	53	1	1	18	2	1	20	1		16	-2	1	
1125	973	73	0	1	14	2	1	20	1		16	2	1	
1126	974	60	1	1	21	1	1	20	1		16	-5	1	
1127	975	73	0	1	17	0	1	20	1		16	-1	1	
1128	976	59	1	1	17	2	1	20	1		19	2	1	
1129	977	60	0	0	18	0	1	20	1		20	2	1	
1130	978	52	1	0	20	0	1	20	1		15	-5	1	
1131	979	75	0	1	11	1	1	20	1		15	4	1	
1132	980	68	1	0	11	0	1	20	1		19	8	0	
1133	981	69	0	1	18	0	1	20	1		18	0	1	
1134	982	54	1	0	19	0	1	20	1		17	-2	1	
1135	983	79	0	0	14	0	1	20	1		19	5	1	
1136	984	32	1	1	20	2	1	20	1		21	1	1	
1137	985	45	0	0	19	0	1	20	1		20	1	1	
1138	986	73	1	0	13	0	1	20	1		19	6	0	
1139	987	66	0	0	18	0	1	20	1		20	2	1	
1140	988	54	0	0	18	1	1	20	1		20	2	1	
1141	989	66	1	0	11	2	1	20	1		16	5	1	
1142	990	51	1	1	12	2	1	20	1		14	2	1	
1143	991	57	1	0	18	1	1	20	1		19	1	1	
1144	992	77	0	0	17	1	1	20	1		19	2	1	
1145	993	76	0	1	16	1	1	20	1		21	5	1	
1146	994	70	1	0	16	0	1	20	1		18	2	1	
1147	995	65	0	0	16	2	1	20	1		21	5	1	
1148	996	62	0	1	9	0	1	20	1		11	2	1	
1149	997	80	1	1	11	1	1	20	1		12	1	1	
1150	998	45	0	1	14	1	1	20	1		18	4	1	
1151	999	29	1	0	12	2	1	20	1		18	6	0	
1152	1000	69	0	1	12	0	1	20	1		15	3	1	
1153	1001	81	1	1	17	0	1	20	1		17	0	1	
1154	1002	66	0	0	13	0	1	20	1		16	3	1	
1155	1003	35	1	1	16	0	1	20	1		20	4	1	
1156	1004	75	1	1	10	1	1	20	1		18	8	0	
1157	1005	62	0	0	13	0	1	20	1		15	2	1	
1158	1006	73	0	1	9	0	1	20	1		17	8	0	
1159	1007	67	1	1	10	0	1	20	1		10	0	1	
1160	1008	67	1	1	16	0	1	20	1		15	-1	1	
1161	1009	81	0	0	10	1	1	20	1		14	4	1	
1162	1010	76	0	1	3	0	1	20	1		11	8	0	
1163	1011	80	0	0	14	0	1	20	1		21	7	0	
1164	1012	62	0	0	18	1	1	20	1		20	2	1	
1165	1013	59	0	1	9	2	1	20	1		15	6	0	
1166	1014	59	0	0	11	2	1	20	1		17	6	0	
1167	1015	79	0	0	8	0	1	20	1		18	10	0	
1168	1016	59	1	0	18	1	1	20	1		21	3	1	
1169	1017	81	0	0	13	0	1	20	1		9	-4	1	
1170	1018	67	1	0	12	1	1	20	1		16	4	1	
1171	1019	76	0	0	12	1	1	20	1		19	7	0	
1172	1020	86	0	0	11	0	1	20	1		13	2	1	
1173	1021	59	0	1	16	0	1	20	1		14	-2	1	
1174	1022	62	1	1	21	1	1	20	1		21	0	1	
1175	1023	73	1	0	13	1	1	20	1		18	5	1	
1176	1024	71	1	0	12	0	1	20	1		14	2	1	
1177	1025	73	0	1	13	2	1	20	1		12	-1	1	
1178	1026	72	0	0	18	1	1	20	1		21	3	1	
1179	1027	88	1	1	18	0	1	20	1		19	1	1	
1180	1028	54	1	1	16	1	1	20	1		18	2	1	
1181	1029	65	1	1	17	0	1	20	1		16	-1	1	
1182	1030	76	1	0	14	0	1	20	1		20	6	0	
1183	1031	66	1	0	14	0	1	20	1		18	4	1	
1184	1032	54	1	1	19	0	1	20	1		20	1	1	
1185	1033	60	1	0	17	0	1	20	1		16	-1	1	
1186	1034	62	0	0	16	1	1	20	1		20	4	1	
1187	1035	65	0	1	14	0	1	20	1		15	1	1	
1188	1036	61	0	1	18	0	1	20	1		17	-1	1	
1189	1037	71	1	1	14	0	1	20	1		19	5	1	
1190	1038	68	1	1	16	0	1	20	1		21	5	1	
1191	1039	82	0	0	5	0	1	20	1		10	5	1	
1192	1040	65	0	1	11	0	1	20	1		15	4	1	
1193	1041	71	1	0	11	0	1	20	1		17	6	0	
1194	1042	64	1	0	18	0	1	20	1		20	2	1	
1195	1043	72	0	1	15	0	1	20	1		20	5	1	
1196	1044	40	1	1	15	0	1	20	1		21	6	0	
1197	1045	57	0	0	16	1	1	20	1		18	2	1	
1198	1046	60	1	1	18	1	1	20	1		20	2	1	
1199	1047	39	0	0	15	0	1	20	1		15	0	1	
1200	1048	61	1	1	4	2	1	20	1		4	0	1	
1201	1049	56	1	0	6	2	1	20	1		16	10	0	
1202	1049	56	1	1	7	2	1	20	1		19	12	0	
1203	1050	81	1	1	17	1	1	20	1		21	4	1	
1204	1051	72	0	0	13	1	1	20	1		14	1	1	
1205	1052	67	1	0	20	1	1	20	1		20	0	1	
1206	1053	57	1	1	11	1	1	20	1		21	10	0	
1207	1054	57	0	1	13	0	1	20	1		15	2	1	
1208	1055	58	0	0	9	0	1	20	1		8	-1	1	
1209	1056	78	0	1	9	0	1	20	1		18	9	0	
1210	1057	58	0	1	19	1	1	20	1		21	2	1	
1211	1058	46	0	1	12	1	1	20	1		15	3	1	
1212	1059	66	1	0	15	0	1	20	1		18	3	1	
1213	1060	46	0	1	17	1	1	20	1		16	-1	1	
1214	1061	42	0	1	14	0	1	20	1		15	1	1	
1215	1062	58	1	1	18	0	1	20	1		17	-1	1	
1216	1063	59	1	1	17	1	1	20	1		20	3	1	
1217	1064	71	0	0	13	0	1	20	1		17	4	1	
1218	1065	61	0	0	12	1	1	20	1		14	2	1	
1219	1066	65	1	1	14	2	1	20	1		17	3	1	
1220	1067	70	1	0	14	0	1	20	1		19	5	1	

1221	1068	57	0	0	13	1	1	20	1		19	6	0		1
1222	1069	47	1	1	16	2	1	20	1		18	2	1		1
1223	1070	66	0	0	13	1	1	20	1		15	2	1		1
1224	1071	61	1	1	12	3	1	20	1		14	2	1		1
1225	1072	63	0	1	17	1	1	20	1		16	-1	1		1
1226	1073	65	0	1	14	0	1	20	1		15	1	1		1
1227	1074	73	0	1	16	0	1	20	1		16	0	1		1
1228	1075	78	1	0	12	0	1	20	1		16	4	1		1
1229	1076	42	1	0	13	1	1	20	1		20	7	0		1
1230	1077	63	1	0	13	0	1	20	1		18	5	1		1
1231	1078	70	0	0	15	0	1	20	1		15	0	1		1
1232	1079	63	0	1	12	0	1	20	1		17	5	1		1
1233	1080	39	0	0	11	2	1	20	1		18	7	0		1
1234	1081	61	1	0	11	0	1	20	1		15	4	1		1
1235	1082	73	1	0	14	0	1	20	1		18	4	1		1
1236	1083	83	1	0	18	0	1	20	1		18	0	1		1
1237	1084	80	0	1	14	1	1	20	1		17	3	1		1
1238	1085	69	1	0	18	0	1	20	1		20	2	1		1
1239	1086	63	0	0	16	1	1	20	1		17	1	1		1
1240	1087	67	1	1	12	2	1	20	1		18	6	0		1
1241	1088	71	1	1	17	0	1	20	1		16	-1	1		1
1242	1089	73	1	1	11	0	1	20	1		14	3	1		1
1243	1090	76	1	1	13	0	1	20	1		15	2	1		1
1244	1091	58	1	0	12	1	1	20	1		15	3	1		1
1245	1092	68	1	0	12	0	1	20	1		14	2	1		1
1246	1093	51	0	1	13	1	1	20	1		17	4	1		1
1247	1094	59	1	1	19	0	1	20	1		18	-1	1		1
1248	1095	43	0	0	17	0	1	20	1		20	3	1		1
1249	1096	60	0	1	15	0	1	20	1		18	3	1		1
1250	1097	73	0	1	13	2	1	20	1		15	2	1		1
1251	1098	83	1	0	19	1	1	20	1		19	0	1		1
1252	1099	81	0	1	19	0	1	20	1		21	2	1		1
1253	1100	64	0	0	17	1	1	20	1		19	2	1		1
1254	1101	48	0	0	13	2	1	20	1		14	1	1		1
1255	1102	68	0	0	13	0	1	20	1		20	7	0		1
1256	1103	76	0	1	18	1	1	20	1		16	-2	1		1
1257	1104	58	0	0	10	0	1	20	1		11	1	1		1
1258	1105	54	1	0	14	0	1	20	1		15	1	1		1
1259	1106	77	0	0	19	0	1	20	1		18	-1	1		1
1260	1107	66	0	1	15	0	1	20	1		17	2	1		1
1261	1108	74	1	0	12	0	1	20	1		13	1	1		1
1262	1109	61	0	0	17	0	1	20	1		20	3	1		1
1263	1110	74	0	0	13	0	1	20	1		14	1	1		1
1264	1111	65	0	1	14	0	1	20	1		19	5	1		1
1265	1112	66	0	1	17	1	1	20	1		20	3	1		1
1266	1113	48	1	0	17	0	1	20	1		18	1	1		1
1267	1114	88	1	0	12	0	1	20	1		13	1	1		1
1268	1115	70	0	1	9	0	1	20	1		19	10	0		1
1269	1116	51	0	0	10	0	1	20	1		14	4	1		1
1270	1117	69	1	0	14	0	1	20	1		15	1	1		1
1271	1118	74	1	1	14	0	1	20	1		14	0	1		1
1272	1119	58	1	0	17	0	1	20	1		17	0	1		1
1273	1120	67	0	1	8	0	1	20	1		15	7	0		1
1274	1121	60	0	1	9	1	1	20	1		14	5	1		1
1275	1122	80	1	1	15	0	1	20	1		16	1	1		1
1276	1123	72	0	0	12	0	1	20	1		17	5	1		1
1277	1124	62	0	0	13	1	1	20	1		15	2	1		1
1278	1125	62	1	1	18	0	1	20	1		16	-2	1		1
1279	1126	45	1	1	17	2	1	20	1		18	1	1		1
1280	1127	67	0	0	10	1	1	20	1		14	4	1		1
1281	1128	79	1	0	14	0	1	20	1		18	4	1		1
1282	1129	17	1	0	16	2	1	20	1		18	2	1		1
1283	1130	64	0	0	18	0	1	20	1		19	1	1		1
1284	1131	54	0	1	19	1	1	20	1		21	2	1		1
1285	1132	64	1	0	17	1	1	20	1		20	3	1		1
1286	1133	68	0	0	12	3	1	20	1		17	5	1		1
1287	1134	62	0	0	13	0	1	20	1		17	4	1		1
1288	1135	65	1	0	19	0	1	20	1		21	2	1		1
1289	1136	75	1	1	18	0	1	20	1		14	-4	1		1
1290	1137	45	1	0	21	0	1	20	1		21	0	1		1
1291	1138	78	1	1	13	1	1	20	1		21	8	0		1
1292	1139	70	1	1	15	0	1	20	1		19	4	1		1
1293	1140	64	0	1	13	1	1	20	1		18	5	1		1
1294	1141	65	1	1	19	2	1	20	1		16	-3	1		1
1295	1142	58	0	0	18	0	1	20	1		21	3	1		1
1296	1143	78	0	0	12	1	1	20	1		18	6	0		1
1297	1144	72	1	0	14	0	1	20	1		16	2	1		1
1298	1145	61	0	0	13	0	1	20	1		12	-1	1		1
1299	1146	73	1	1	12	0	1	20	1		16	4	1		1
1300	1147	71	0	1	11	0	1	20	1		16	5	1		1
1301	1148	37	0	0	10	3	1	20	1		11	1	1		1
1302	1149	83	1	1	12	0	1	20	1		15	3	1		1
1303	1150	65	1	0	12	0	1	20	1		17	5	1		1
1304	1151	66	0	0	17	1	1	20	1		19	2	1		1
1305	1152	15	0	0	12	2	1	20	1		13	1	1		1
1306	1153	69	1	0	15	1	1	20	1		15	0	1		1
1307	1154	85	1	0	9	0	1	20	1		15	6	0		1
1308	1155	65	1	0	14	0	1	20	1		16	2	1		1
1309	1156	82	0	1	9	2	1	20	1		11	2	1		1
1310	1157	79	1	1	13	0	1	20	1		19	6	0		1
1311	1158	79	0	0	15	0	1	20	1		21	6	0		1
1312	1159	24	1	1	19	1	1	20	1		19	0	1		1
1313	1160	48	1	0	17	0	1	20	1		16	-1	1		1
1314	1161	67	0	0	16	0	1	20	1		19	3	1		1
1315	1162	76	1	0	16	0	1	20	1		18	2	1		1
1316	1163	76	0	1	12	0	1	20	1		18	6	0		1
1317	1164	81	1	0	17	0	1	20	1		20	3	1		1
1318	1165	46	1	1	11	0	1	20	1		20	9	0		1
1319	1166	74	0	1	18	1	1	20	1		18	0	1		1
1320	1167	66	1	0	15	0	1	20	1		19	4	1		1
1321	1168	73	0	1	15	0	1	20	1		17	2	1		1
1322	1169	63	0	0	15	0	1	20	1		16	1	1		1
1323	1170	62	0	0	10	1	1	20	1		17	7	0		1
1324	1171	57	0	0	12	1	1	20	1		19	7	0		1
1325	1172	49	1	1	16	0	1	20	1		16	0	1		1
1326	1173	60	0	0	13	1	1	20	1		15	2	1		1
1327	1174	56	1	0	13	0	1	20	1		15	2	1		1
1328	1175	21	0	1	10	0	1	20	1		17	7	0		1
1329	1176	73	1	0	18	0	1	20	1		19	1	1		1
1330	1177	87	0	0	15	3	1	20	1		19	4	1		1
1331	1178	69	1	0	16	2	1	20	1		19	3	1		1
1332	1179	33	1	0	14	2	1	20	1		16	2	1		1
1333	1180	61	1	0	13	0	1	20	1		16	3	1		1
1334	1181	87	0	0	14	0	1	20	1		20	6	0		1
1335	1182	44	1	1	12	2	1	20	1		15	3	1		1
1336	1183	63	1	0	14	0	1	20	1		19	5	1		1
1337	1184	54	0	1	20	3	1	20	1		21	1	1		1
1338	1185	78	1	1	12	0	1	20	1		11	-1	1		1
1339	1186	92	0	1	9	0	1	20	1		14	5	1		1
1340	1187	70	0	1	15	0	1	20	1		13	-2	1		1
1341	1188	64	1	1	12	0	1	20	1		21	9	0		1
1342	1189	65	0	0	15	0	1	20	1		14	-1	1		1
1343	1190	64	0	0	11	0	1	20	1		17	6	0		1
1344	1191	65	1	1	16	0	1	20	1		20	4	1		1
1345	1192	71	1	1	14	0	1	20	1		14	0	1		1
1346	1193	57	1	1	17	0	1	20	1		18	1	1		1
1347	1194	73	0	1	20	1	1	20	1		19	-1	1		1
1348	1195	60	1	0	12	2	1	20	1		18	6	0		1
1349	1196	27	1	1	12	2	1	20	1		17	5	1		1
1350	1197	97	1	0	10	0	1	20	1		10	0	1		1
1351	1198	70	1	1	16	2	1	20	1		18	2	1		1
1352	1199	24	1	0	12	3	1	20	1		17	5	1		1
1353	1200	64	0	0	16	2	1	20	1		20	4	1		1
1354	1201	62	1	1	16	0	1	20	1		16	0	1		1
1355	1202	62	1	0	16	0	1	20	1		21	5	1		1
1356	1203	71	0	0	14	0	1	20	1		18	4	1		1
1357	1204	83	1	0	16	0	1	20	1		18	2	1		1
1358	1205	69	0	0	16	1	1	20	1		18	2	1		1
1359	1206	81	0	0	11	0	1	20	1		18	7	0		1
1360	1207	64	0	1	15	2	1	20	1		18	3	1		1
1361	1208	70	0	1	19	1	1	20	1		19	0	1		1
1362	1209	73	0	0	13	0	1	20	1		19	6	0		1
1363	1210	45	0	0	15	1	1	20	1		19	4	1		1
1364	1211	85	1	0	18	0	1	20	1		20	2	1		1
1365	1212	73	0	0	13	1	1	20	1		18	5	1		1
1366	1213	92	1	0	17	0	1	20	1		19	2	1		1
1367	1214	63	0	0	19	1	1	20	1		18	-1	1		1
1368	1215	64	1	1	14	1	1	20	1		17	3	1		1
1369	1216	68	0	0	13	0	1	20	1		17	4	1		1
1370	1217	86	1	1	9	2	1	20	1		15	6	0		1
1371	1218	83	1	1	7	1	1	20	1		13	6	0		1
1372	1219	62	0	0	14	1	1	20	1		18	4	1		1
1373	1220	66	0	1	18	2	1	20	1		18	0	1		1
1374	1221	67	1	0	18	0	1	20	1		20	2	1		1
1375	1222	65	0	0	18	1	1	20	1		19	1	1		1
1376	1223	79	0	0	6	0	1	20	1		9	3	1		1
1377	1224	40	0	0	14	2	1	20	1		19	5	1		1
1378	1225	83	1	1	8	1	1	20	1		10	2	1		1
1379	1226	64	0	1	12	1	1	20	1		18	6	0		1
1380	1227	80	1	0	15	0	1	20	1		20	5	1		1
1381	1228	65	0	0	18	1	1	20	1		16	-2	1		1
1382	1229	65	1	1	14	1	1	20	1		18	4	1		1
1383	1230	62	1	1	16	2	1	20	1		19	3	1		1
1384	1231	66	0	1	14	0	1	20	1		21	7	0		1
1385	1232	62	1	1	18	0	1	20	1		20	2	1		1
1386	1233	47	0	0	12	0	1	20	1		20	8	0		1
1387	1234	78	1	0	15	2	1	20	1		13	-2	1		1
1388	1235	58	1	1	13	2	1	20	1		19	6	0		1
1389	1236	64	1	1	13	0	1	20	1		17	4	1		1
1390	1237	64	0	0	17	0	1	20	1		21	4	1		1
1391	1238	67	0	1	16	0	1	20	1		19	3	1		1
1392	1239	60	1	0	18	2	1	20	1		18	0	1		1
1393	1240	71	0	1	18	0	1	20	1		17	-1	1		1
1394	1241	73	1	0	15	0	1	20	1		20	5	1		1
1395	1242	85	1	0	13	0	1	20	1		19	6	0		1
1396	1243	14	1	0	10	2	1	20	1		21	11	0		1
1397	1244	57	0	1	13	2	1	20	1		19	6	0		1
1398	1245	72	1	1	16	0	1	20	1		15	-1	1		1
1399	1246	85	0	1	15	0	1	20	1		17	2	1		1
1400	1247	66	1	0	16	1	1	20	1		20	4	1		1
1401	1248	64	1	0	18	0	1	20	1		20	2	1		1
1402	1249	73	0	0	14	1	1	20	1		21	7	0		1
1403	1250	76	0	1	13	0	1	20	1		19	6	0		1
1404	1251	71	0	0	14	0	1	20	1		19	5	1		1
1405	1252	72	1	0	15	1	1	20	1		20	5	1		0
1406	1252	72	1	1	16	1	1	20	1		18	2	1		0
