# Supplementary material for: Intraocular pressure elevation after subtenon triamcinolone acetonide injection; Multicentre retrospective cohort study in Japan
Source: PLoS One. 2019 Dec 5;14(12):e0226118. doi: 10.1371/journal.pone.0226118 (PMC6894825; doi:10.1371/journal.pone.0226118)
Supplement: Attachment — Submitted filename: Response to Reviewers_20191111.docx [file pone.0226118.s002.docx]

Referee(s)' Comments to Author:

Reviewer #1: This paper is important since it addresses the real possibility of iatrogenic disease. However, many issues remain,

1. Abstract.

a. Results: Higher incidence of DME and uveitis are listed as risk factors for raised IOP. Yet in the next line it is mentioned that low incidence of DME is associated with raised IOP. Please clarify

***Response:***

***Younger age, lower baseline IOP, steroid dose, and higher incidence of DME were associated with raised IOP. However, when we fixed the steroid dose at 20 mg, we found that a lower incidence of DME was associated with raised IOP (only when the steroid dose was fixed as mentioned here), suggesting that IOP might increase in association with the steroid dose, especially in patients with DME. Please note that we mentioned this in the Discussion section in the original manuscript.***

2. Methods

a. Would be interesting to know how many patients had received STTA in the study period prior to exclusion due to the criteria given.

***Response:***

***We thank the reviewer for this important comment. Unfortunately, we could not determine the total number of enrolled patients prior to exclusion, because each institution only provided data from patients after exclusion had been performed.***

b. What were the specific diagnosis of all subjects reeving SSTA?

***Response:***

***We mentioned causative diseases in the original manuscript. In the revised version, we have added the following additional details: ‘Causative diseases were DME (632 eyes, 45.0%), RVO (457 eyes, 32.5%), uveitis (223 eyes, 15.9%), and others (94 eyes, 6.7%), including optic neuritis, Thyroid-associated ophthalmopathy, and Irvine-Gass Syndrome’.***

c. Exclusion criteria were exclusion from SSTA or exclusion from the study. Please clarified.

***Response:***

***These were exclusion criteria for the present study. We have added the following text (highlighted in yellow) in the Material and Methods section: ‘Exclusion criteria for this study were: 1) existing glaucoma diagnosis and associated medication; 2) baseline IOP > 21 mmHg; 3) any intraocular surgery except glaucoma surgery within 6 months after STTA injection. A total of 1406 eyes were analysed in 1252 Japanese patients who received STTA.’***

3. Results

a. It would be interesting to know what was the IOP elevation in each disease group

***Response:***

***In accordance with the reviewer’s comment, we have analysed the IOP values in each disease group and added these in the Results section as follows: ‘The highest IOP values in patients with DME, RVO, uveitis, and others were 18.2 ± 4.5 mmHg, 18.1 ± 5.4 mmHg, 18.1 ± 5.7 mmHg, and 17.3 ± 4.8 mmHg, respectively.’***

b. In 23% of cases, the IOP rose > 6 mmHg. How were they all treated? What was the disc like?

***Response:***

***We included this information in the Results section and Table 3 in the original manuscript, as follows: ‘For treatments in patients with IOP increase ≥ 6 mmHg, eye drops were administered in 95 eyes (29.0% of eyes with IOP increase > 6 mmHg, 6.8% of all eyes) and glaucoma surgery was performed in two eyes (1.2% of eyes with IOP increase ≥ 6 mmHg, 0.14% of all eyes).’***

c. In how many did the IOP return back to normal? Did the bolus require to be removed?

***Response:***

***All instances of IOP elevation were resolved after treatment. We have added the following explanation in the Results section: ‘After treatment, IOP returned to normal in patients who had exhibited IOP elevation.’ and ‘After treatment, IOP returned to normal in patients who had exhibited IOP increase ≥ 6 mmHg.’.***

d. Could not find a correlation table in your manuscript to say the IOP is correlated to the underlying disease. A regression analysis would be an appropriate tool.

***Response:***

***We thank the reviewer for this important suggestion. IOP is a continuous variable, whereas causative diseases were represented by a discrete variable. We used Fisher’s exact test for analyses of the association between IOP and causative diseases. Therefore, we calculated p-values as shown in Tables 1 – 4.***

Please address all correspondence to:

Hiroto Ishikawa

Department of Ophthalmology, Hyogo College of Medicine

1-1, Mukogawa-cho, Nishinomiya, Hyogo 663-8501, Japan

Tel; +81-798-45-6462, Fax; +81-798-45-6464, e-mail; ohmyeye@gmail.com

Thank you for your time in reviewing our re-submission. We look forward to hearing from you at your earliest convenience.

Yours sincerely,

Hiroto Ishikawa
